# Cascading One-Pot Synthesis of Biodegradable Uronic Acid-Based Surfactants from Oligoalginates, Semi-Refined Alginates, and Crude Brown Seaweeds

**DOI:** 10.3390/molecules28135201

**Published:** 2023-07-04

**Authors:** Freddy Pessel, Guillaume Noirbent, Cédric Boyère, Sacha Pérocheau Arnaud, Tiphaine Wong, Laura Durand, Thierry Benvegnu

**Affiliations:** 1 SurfactGreen, 11 Allée de Beaulieu, CS 50837, F-35708 Rennes, France; freddy.pessel@surfactgreen.com (F.P.); cedric.boyere@surfactgreen.com (C.B.); 2 CNRS, ISCR-UMR 6226, Ecole Nationale Supérieure de Chimie de Rennes, University of Rennes, F-35000 Rennes, France; guillaume.noirbent@ensc-rennes.fr (G.N.); sacha.spa@gmail.com (S.P.A.); tiphaine.wong@ensc-rennes.fr (T.W.); laura.durand@ensc-rennes.fr (L.D.)

**Keywords:** oligomannuronate, oligoguluronate, oligoalginate, semi-refined alginate, brown seaweed, one pot cascade process, uronic acid-based surfactants, readily biodegradability, non-ecotoxicity

## Abstract

The present article describes a one-pot and cascade mode process using biocompatible/biodegradable reagents, for simply obtaining surfactant compositions comprising mixtures of d-mannuronic acid and l-guluronic acid directly from oligoalginates or semi-refined alginates (mixtures of alginate, cellulose, hemicellulose, laminaran, and fucan). Simple treatments of partial purification of the reaction crudes (elimination of the salts and/or the residual fatty alcohols) or isolation of the surfactant compositions result in sugar-based compounds having performance levels appropriate to applications in detergency. In addition, the challenging extension of this cascading one-pot synthesis technology to crude milled brown seaweeds was successfully carried out to provide promising surface-active compositions made up of alkyl uronate and alkyl glycoside monosaccharides.

## 1. Introduction

Currently, 100% biobased surfactants containing both a hydrophobic tail and a hydrophilic head of plant origin represent 5% of the total surfactants in the market worldwide [1]. This is the most dynamic segment, with growth of more than 20% per year and a high penetration rate in Europe. The introduction of increasingly strict regulatory standards relating to environmental impact and effects on human health should open up new opportunities for these products. On the other hand, the increased demand of consumers for more environmentally friendly products is leading industries to use surfactants or products certified by a label (Ecolabel, COSMOS) which guarantees compliance with this requirement.

Fully 100% biobased surfactants on the market mainly include products derived from sugars, which belong to the non-ionic family. They incorporate a glucose head for alkyl polyglucosides (APGs), sucrose for sucro-esters, and sorbitan for sorbitan esters [2,3,4]. Depending on the number of carbon atoms making up the lipophilic chain (4 to 22), these surfactants may have hydrotropic, foaming, degreasing, wetting, foam- and viscosity-boosting, and emulsifying properties, and may lead to good sensory properties. Anionic sugar-based surfactants are present on the market to a much smaller extent compared with their non-ionic homologs. However, anionic surfactants are currently the most used types, being incorporated in the majority of detergent and cleaning-product formulas in daily use. Their most prominent representatives are linear alkylbenzene sulfonates (LAS), alcohol ether sulfates (AES), secondary alkane sulfonates (SAS), and alcohol sulfates (AS) which are generally highly irritating and ecotoxic [5]. An alkyl polyglucoside carboxylate, Plantapon LGC Sorb (INCI name sodium lauryl glucose carboxylate (and) lauryl glucoside) has been introduced onto the market by Cognis as a new anionic surfactant for applications in body care formulations [4]. An industrial process based on the reaction of sodium monochloroacetate in an aqueous solution of alkyl polyglycoside (without additional solvent) has been developed in this context. Nevertheless, these marketed products do not fulfill all the functions of the non-ionic surfactants derived from ethylene oxide, and even less those of the anionic surfactants which represent the most important part of the detergent market. On the other hand, the cost of renewable raw materials and the relative complexity of the manufacturing processes lead to an additional cost compared to traditional surfactants. There is a great need for creativity in transforming existing raw materials and developing new ones [6,7,8,9,10,11,12,13,14], with a certain number of technological obstacles to be overcome: the high oxygen content of plant-based products, which makes chemical transformation more difficult to achieve, the difficulty in identifying commercially available natural anionic substrates that can be easily transformed into the corresponding anionic surfactants, etc.

The use of starting materials based on algal polysaccharides, which are characterized by an original chemical functionality compared with polysaccharides from terrestrial plants, constitutes an approach that could make it possible to broaden the fields of application of the 100% biobased surfactants. Alginates, which are polysaccharides present in the cell wall of brown algae, are produced on a scale of 30,000 tons worldwide; their use has several advantages from the point of view of environmental impact, since their production does not require agricultural land, fresh water, or fertilizers and phytosanitary products [15]. These polysaccharides are formed by (1-4) glycosidic bonds between the two monomers, d-mannuronate (M) and its C-5 epimer l-guluronate (G). These M and G units are arranged irregularly, by homopolymeric blocks (MM or GG) separated by heterogenous blocks of both uronates (MG) along the alginate chain. Some studies have already shown the possibility of exploiting d-mannuronate oligomers in the surfactant field. However, the preparation of compositions based on monomeric mixtures of both l-guluronate and d-mannuronate units, allowing the valorization of all the sugars present in the biopolymer structure, has not been developed to date.

Alkyl d-mannopyranosiduronate surfactants have been produced from d-mannuronic acid oligomers through one-pot acid glycosidic bond hydrolysis, esterification, and stereocontrolled Fisher glycosylation in butanol followed by transesterification/transglycosylation processes in fatty alcohols [16]. These non-ionic surfactants have identical lipophilic chains and can subsequently be saponified in order to obtain single-tailed anionic surfactants with a carboxylic acid unit. These oligomannuronate-derived amphiphiles exhibit attractive surface tension and foaming properties. However, the exclusive use of mannuronate oligomers as starting materials for the production of anionic uronate surfactants is very restrictive and the development of new processes allowing for valorization of the entire alginate and possibly additional polysaccharides present in the algal cell wall is essential to facilitate the industrial transfer of these innovative products.

**Figure 1:**
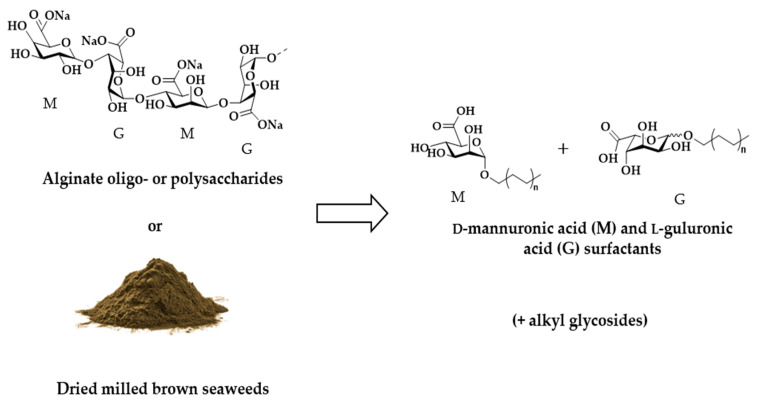
Transformation of poly(oligo)alginates or dried milled brown seaweeds into d-mannuronic acid and l-guluronic acid-based surfactant compositions.

Within this context, we investigated a novel strategy to synthesize surfactant compositions directly from less refined starting materials to reduce costs and improve the properties expected in the surfactants field (Figure 1). The first challenge was to identify reaction conditions that enable the one-pot solvent-free transformation of oligoalginates (composed of both M and G units) or semi-refined alginates (mixtures mainly composed of alginate, cellulose, and fucan) into compositions that contain monomeric surfactants in the form of the two uronic acids (l-guluronic acid and d-mannuronic acid), and hexoses and pentoses derived from other polysaccharides present in the algal extract (semi-refined alginates). In addition, our objective was to propose an eco-friendly process for the direct conversion of raw algae into *n*-alkyl glycosiduronic acids and *n*-alkyl glycosides without the use of organic solvents, i.e., by simultaneously carrying out the steps of extraction, depolymerization by acid hydrolysis with a minimum of water, and esterification/glycosylation. The carboxylic acid-based surfactant compositions produced will be evaluated in terms of surface activities, biodegradability, and ecotoxicity.

## 2. Results

### 2.1. Preparation of Starting Materials

Poly(oligo)mannuronates and poly(oligo)guluronates are attractive raw materials for the development of original 100% biobased surfactants. They are obtained from fresh or dry algae derived from *Laminaria digitata* using a process based on a pre-extraction of the alginates, followed by several steps of precipitation by modulating the pH of the reaction medium in order to separate the G blocks and the M blocks constituting the alginate [16,17]. Finally, an acid hydrolysis step affords the oligomannuronate (DP = 4: Table 1) or oligoguluronate (DP = 30: Table 1).

Concerning oligoalginate (OAlg) and semi-refined alginate (s-r Alg) [18], the procedure involves acid leaching of fresh or dry algae derived from *Laminaria digitata*, followed by dissolution of the sodium alginates by increasing the pH of the medium. Then, a solid/liquid separation is performed in order to remove the algal residues. At this stage, the liquid fraction can be freeze-dried and constitutes the semi-refined alginates (Table 1) in the form of sodium alginates. In order to obtain refined oligoalginates, a purification step is introduced into the previous steps. After separation of the algal residues, the latter purification step includes or consists of precipitation of the alginic acid by reducing the pH, followed by several washes with acidic water in order to remove the co-products. Increasing the pH with Na_2_CO_3_ makes it possible to dissolve the sodium alginates again while limiting the salt, compared with the use of sodium hydroxide. Finally, the alginate solution is treated with acid in order to reduce the degree of polymerization, which produces, after a step of freezing and then freeze-drying, the oligoalginate of DP 12.7 (Table 1).

Regarding the dried milled brown seaweeds, Asco T10 from the *Ascophyllum nodosum* species (Thorverk, Westfjords, Iceland) was used.

### 2.2. Synthesis from Oligoalginates

In order to achieve the goal of synthesizing novel biobased uronic surfactants from alginates, several uronate sources have been investigated to assess their potential as biomass feedstock for the preparation of such amphiphilic molecules through green synthetic pathways. At first, oligomannuronate (OM) and oligoguluronate (OG) were used in order to define the optimal reaction conditions for the one-pot cascade synthesis of uronate monomeric surfactants. Then the optimized conditions were transposed to oligoalginate with a M/G ratio of 1.4 and a DP of 12.7.

#### 2.2.1. Synthesis from Oligomannuronate (OM)

**Scheme 1:**
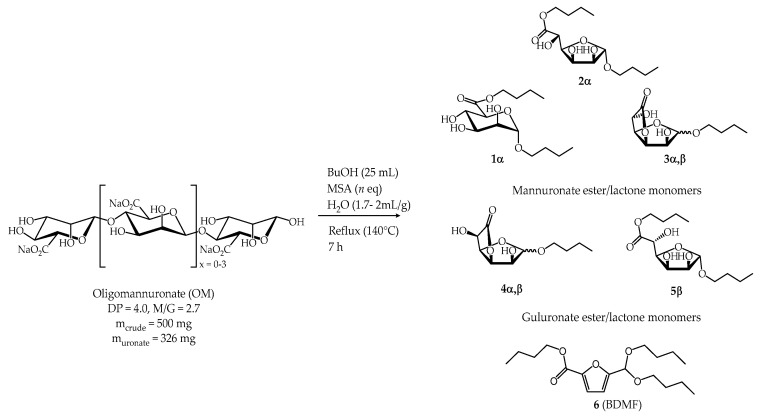
Synthesis of butylated mannuronate monomers **1**–**3** in addition to side-product **6** (BDMF) and butylated guluronate monomers **4**–**5**.

In the first stage of the study, the reactivity of sodium oligomannuronate (M/G = 2.7, DP = 4, mass = 500 mg) was investigated towards acid hydrolysis, esterification, and Fisher glycosidation conditions. Acid treatment of OM was performed for 7 h with various amounts of methane sulfonic acid (MSA) in the presence of water and butanol (Scheme 1). Based on previous results obtained in the laboratory [16], the volume of butanol was set at 25 mL. The use of larger quantities of butanol did not allow a significant improvement of the yield while presenting a problem of oversizing the production facilities. However, whereas the reactions were previously envisaged in dry butanol [16], in these new experiments it was found that the solubility of the oligomannuronate was low, thus preventing the reactions from carrying out. The addition of water at a rate of 1.7–2 mL/g of algal extract proved to be sufficient for the solubilization of the oligomannuronate in the reaction mixture. The butanol was distilled out during heating and the water initially added to the reaction mixture and formed during the reaction was eliminated using a Dean-Stark apparatus (140 °C). After neutralization and workup, the organic residue was purified using silica gel column chromatography to isolate fractions enriched in a few compounds, which facilitated the identification of the glycoside-esters present in the mixture by ^1^H NMR studies (Figures S1–S4, Supplementary Materials). Indeed, ^1^H NMR analysis led to the identification of characteristic signals for each compound, as shown in Table 2. Thus, the formation of (*n*-butyl) *n*-butyl α-d-mannopyranosiduronate **1α** was observed as the major monosaccharide product (52 mol%, isolated as a pure fraction, 33% yield, Table 3) in addition to a mixture of isomers: (*n*-butyl) *n*-butyl α-d-mannofuranosiduronate **2α** (7 mol%), *n*-butyl α-d-mannofuranosidurono-6,3-lactone **3α** (11 mol%), and *n*-butyl β-d-mannofuranosidurono-6,3-lactone **3β** (13 mol%). Products from the guluronate units present in smaller quantities in the raw material (M/G ratio = 2.7) were also identified, i.e., *n*-butyl-α-l-gulofuranosidurono-6.3-lactone **4α** (5 mol%), *n*-butyl-β-l-gulofuranosidurono-6.3-lactone **4β** (5 mol%), and (*n*-butyl) *n*-butyl-β-l-gulofuranosiduronate **5β** (3 mol%). Alongside these uronate derivatives, a side product **6** was isolated, butyl-5-(dibutoxymethyl)-2-furoate (BDMF) (5 mol%), and it was characterized by NMR (Scheme 1). This furan compound results from the dehydration of mannuronate/guluronate esters and lactone monomers (named butyl uronates) as reported in earlier studies [19]. The total amount of the butylated Man and Gul derivatives **1**–**5** is quite satisfactory but it was noticed that the increase in MSA quantity would further degrade the butyl uronates (Table 3). The overall yield could not be calculated as the isomers possess different molecular weights. It should be noted that 99% or 70% MSA can be used indiscriminately since the same results were obtained in both cases.

**Scheme 2:**
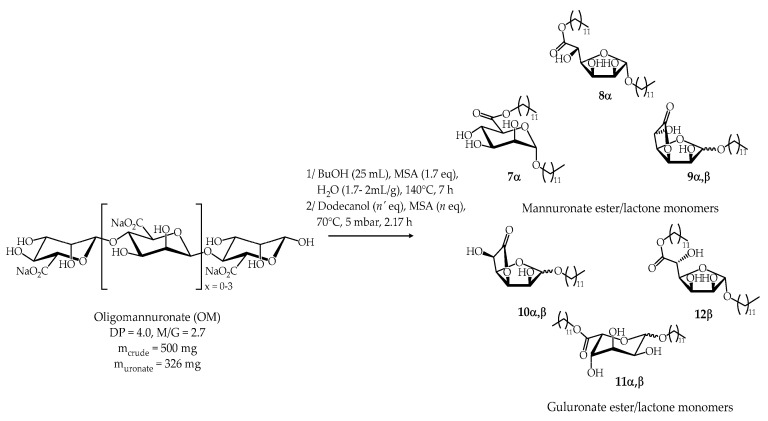
Synthesis of dodecyl mannuronate **7**–**9** and guluronate **10**–**12** ester/lactone monomers.

Next, seeing the possible transformation of oligomannuronates into monomeric derivatives, the optimal conditions (1.7 eq MSA, BuOH (25 mL), H_2_O (1.7–2 mL/g of crude OM), 140 °C, 7 h) were reproduced and the reaction mixture containing the butyl ester glycoside products **1**–**5** was subjected directly to simultaneous transesterification and transglycosylation with dodecanol (DodOH). Then the replacement of the butyl chains by the C_12_ chains was performed in situ (Scheme 2) under reduced pressure (5 mbar) at 70 °C for 2 h 10 m after the addition of an extra equivalent of MSA (99% or 70%). The reduced pressure allows the removal of the butanol and shifts the equilibrium towards the transesterification and transglycosylation products. A variety of reaction conditions with different quantities of dodecanol and MSA were envisaged (Table 4). After a workup of the medium, two successive purifications by column chromatography were performed. The first one allowed the isolation of the mixture of uronate monomers and the second one aimed at obtaining fractions enriched in a few compounds, which facilitated the identification of the glycoside-esters present in the mixture by ^1^H NMR studies. After those purification steps, (*n*-dodecyl) *n*-dodecyl α-d-mannopyranosiduronate **7α** was isolated in addition to several fractions containing mixtures of isomers corresponding to (*n*-dodecyl) *n*-dodecyl α-d-mannofuranosiduronate **8α,** *n*-dodecyl α,β-d-mannofuranosidurono-6,3-lactone **9α,β**, *n*-dodecyl-α,β-l-gulofuranosidurono-6.3-lactone **10α,β**, (*n*-dodecyl) *n*-dodecyl-α,β-l-gulopyranosiduronate **11α,β**, and (*n*-dodecyl) *n*-dodecyl-β-l-gulofuranosiduronate **12β** (Scheme 2) (Figures S5–S8, Supplementary Materials).

The comparison of entries 1 and 2 (Table 4) indicates that the use of dodecanol in large excess does not improve the yield. Finally, the comparison of entries 2 and 3 shows that the decrease in the number of equivalents of MSA introduced for the second step disadvantages the formation of **7α**. The reaction conditions of entry 2 (4 eq DodOH, 1 eq MSA) that provided dodecyl mannuronate **7α** with an overall yield of 25% were thus selected. The molar composition of the mannuronate and guluronate mixture was determined by integrating the characteristic ^1^H NMR signals of each compound of the uronate mixture (Table 5) resulting from the first column chromatography (Figures S5 and S6, Supplementary Materials): (*n*-dodecyl) *n*-dodecyl α-d-mannopyranosiduronate **7α** (48 mol%), (*n*-dodecyl) *n*-dodecyl α-d-mannofuranosiduronate **8α** (9 mol%), *n*-dodecyl α-d-mannofuranosidurono-6,3-lactone **9α** (5 mol%), *n*-dodecyl β-d-mannofuranosidurono-6,3-lactone **9β** (22 mol%), *n*-dodecyl-α-l-gulofuranosidurono-6.3-lactone **10α** (3 mol%), *n*-dodecyl-β-l-gulofuranosidurono-6.3-lactone **10β** (3 mol%), (*n*-dodecyl) *n*-dodecyl-β-l-gulopyranosiduronate **11β** (5 mol%), and (*n*-dodecyl) *n*-dodecyl-β-l-gulofuranosiduronate **12β** (6 mol%). It is noteworthy that (*n*-dodecyl) *n*-dodecyl-α-l-gulopyranosiduronate **11α** was not detectable in this mixture due to its presence in quantities that were too small.

**Scheme 3:**
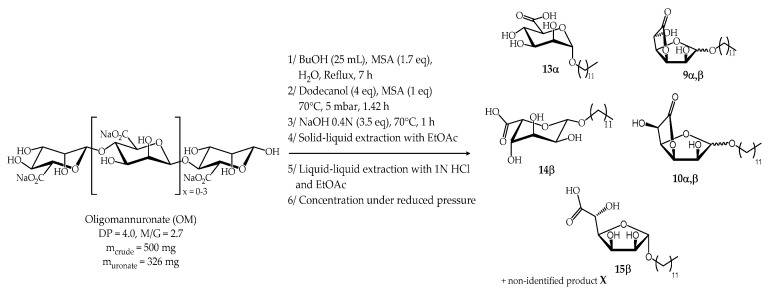
Composition of one-pot and cascade-mode synthesized **H**–**C_12_ Man**-based surfactant.

The next challenge was to develop a one-pot three-step cascade for the synthesis of uronate surfactants possessing a single alkyl chain (Scheme 3). For this purpose, saponification (0.4 N NaOH, 3 eq, 70 °C, 1 h) was carried out directly in the reaction mixture obtained after the transesterification/transglycosylation step. Removal of dodecanol was performed at the end of the process through the removal of water by freeze-drying and the addition of EtOAc into the reaction medium followed by a filtration step. A precipitate composed of the uronate derivatives and additional salts was isolated whereas the filtrate contained the entire fatty alcohol. This solid fraction containing the desired products consists of 96–97% dry matter and 36–37% mineral matter for repeated runs (thermogravimetric analyses). The large amount of mineral matter comes from the raw material used, which already contained it (31.9% dry/crude), and from the NaOH used for the saponification reaction. As the significant amount of mineral matter present in the sodium uronate composition is likely to modify the properties of the surfactants, a purification method allowing for their removal was achieved through liquid-liquid extraction using a 1N HCl aqueous phase and EtOAc. After concentrating the organic phase under reduced pressure, the mannuronate and guluronate surfactants were isolated as carboxylic acids and lactones. ^1^H NMR analysis revealed the presence of *n*-dodecyl α-d-mannopyranosiduronic acid **13α** (47 mol%**)**, *n*-dodecyl α-d-mannofuranosidurono-6,3-lactone **9α** (4 mol%), *n*-dodecyl β-d-mannofuranosidurono-6,3-lactone **9β** (12% molar), *n*-dodecyl β-l-gulopyranosiduronic acid **14β** (4 mol%**)**, *n*-dodecyl α-l-gulofuranosidurono-6,3-lactone **10α** (9 mol%), *n*-dodecyl β-l-gulofuranosidurono-6,3-lactone **10β** (9 mol%), and *n*-dodecyl-β-l-gulofuranosiduronic acid **15β** (4 mol%), in addition to a non-identified product **X** (~10 mol%) which could correspond to *n*-dodecyl d-mannofuranosiduronic acid (Figures S9 and S10, Supplementary Materials). ^1^H NMR analysis led to the identification of characteristic signals for each compound, as shown in Table 6. The surface tension measurements obtained with this **H**–**C_12_ Man** composition are presented in Section 2.5.

#### 2.2.2. Synthesis from Oligoguluronate (OG)

**Scheme 4:**
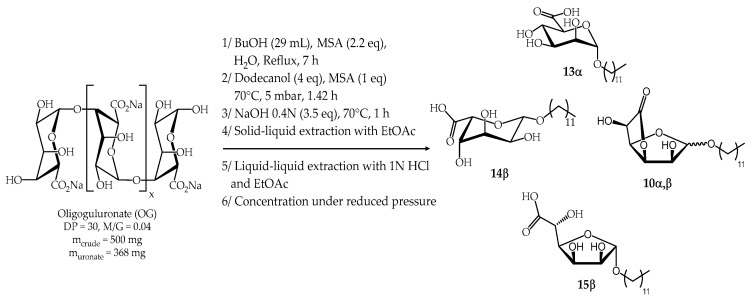
One-pot and cascade-mode synthesis of the **H**–**C_12_ Gul**-based surfactant and its composition.

As the alginate biomass selected either oligoalginates or semi-refined alginates, which are composed of both mannuronate and guluronate units, the previously developed synthetic pathway had to be tested from oligoguluronate (OG) as well. It is noteworthy that more MSA (2.2 eq) was required (Scheme 4) for OG, probably due to the α-binding between guluronate units and/or their higher DP. The same workup based on a solid-liquid extraction step with EtOAc was performed to eliminate dodecanol. This uronate-based mixture was then acidified and purified by liquid-liquid extraction in order to obtain the H-C_12_ derivatives and remove the salts. ^1^H NMR analysis revealed the presence of the following products: *n*-dodecyl α-d-mannopyranosiduronic acid **13α** (15 mol%**)**, *n*-dodecyl β-l-gulopyranosiduronic acid **14β** (13 mol%**)**, *n*-dodecyl α-l-gulofuranosidurono-6,3-lactone **10α** (26 mol%), *n*-dodecyl β-l-gulofuranosidurono-6,3-lactone **10β** (26 mol%), and *n*-dodecyl-β-l-gulofuranosiduronic acid **15β** (21 mol%). The presence of *n*-dodecyl α,β-d-mannofuranosidurono-6,3-lactone **9α,β** was also observed in trace amounts but its quantification was not possible, unlike in the case of synthesis from oligoalginate (See Section 2.2.3). ^1^H NMR analysis led to the identification of characteristic signals for each compound, as shown in Table 7 (Figures S11 and S12, Supplementary Materials). The surface tension measurements obtained with this **H**–**C_12_ Gul** composition are presented in Section 2.5.

#### 2.2.3. Synthesis from Oligoalginate (OAlg)

**Scheme 5:**
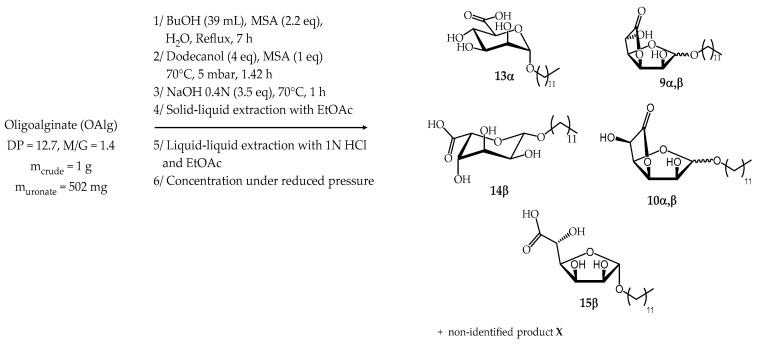
One-pot and cascade-mode synthesis of **H**–**C_12_ OAlg** surfactant composition.

As the process has been shown to be applicable to both d-mannuronate and l-guluronate units, the same synthetic pathway has been tested with oligoalginate (OAlg) composed of d-Man and l-Gul units with the same reaction conditions used for oligoguluronate (OG) (Scheme 5). The OAlg starting raw material is economically advantageous as less purification is required in comparison to OM and OG. The mixture obtained after the saponification step contains 93.4% dry matter and 41.8% mineral matter. The higher percentage of mineral matter compared to oligomannuronate and oligoguluronate is explained by the higher mineral content of the raw material (44.3% dry/crude). Further acidic treatment of the reaction medium was performed as developed for the OM and OG raw materials (Scheme 5). The final **H**–**C_12_ OAlg** surfactant composition is characterised by the presence of *n*-dodecyl α-d-mannopyranosiduronic acid **13α** (23 mol%), *n*-dodecyl α-d-mannofuranosidurono-6,3-lactone **9α** (4 mol%), *n*-dodecyl β-d-mannofuranosidurono-6,3-lactone **9β** (9 mol%), *n*-dodecyl β-l-gulopyranosiduronic acid **14β** (9 mol%), *n*-dodecyl α-l-gulofuranosidurono-6,3-lactone **10α** (20 mol%), *n*-dodecyl β-l-gulofuranosidurono-6,3-lactone **10β** (20 mol%), and *n*-dodecyl-β-l-gulofuranosiduronic acid **15β** (9 mol%) (Table 8). The presence of the non-identified product **X** (6 mol%) formed during the one-pot synthesis of the **H**–**C_12_ Man** composition was also observed. ^1^H NMR analysis led to the identification of characteristic signals for each compound, as shown in Table 8 (Figures S13 and S14, Supplementary Materials). The surface tension measurements obtained with this **H**–**C_12_ OAlg** composition are presented in Section 2.5.

### 2.3. Synthesis from Semi-Refined Alginate (s-r Alg)

**Scheme 6:**
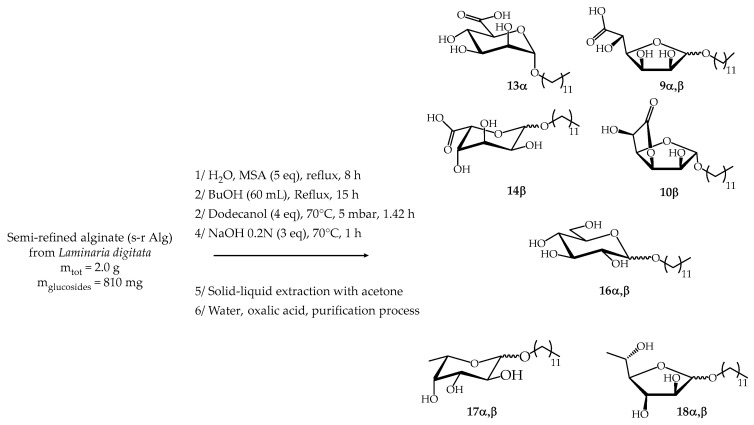
One-pot and cascade-mode synthesis of **H**–**C_12_ s-r Alg** surfactant composition.

In order to further reduce the cost of surfactant production, the process was applied to semi-refined alginate (**s-r Alg**) extracted from the *Laminaria digitata* species composed of d-mannuronate and l-guluronate units in addition to neutral l-fucose, d-glucose, and d-xylose sugars. The first step of the synthetic scheme developed for oligosaccharides could not be directly applied. Indeed, modifications had to be made to take into account the low solubility of the mixture in butanol due to the high degree of polymerization of the polysaccharides. Thus, the synthesis of the butyl C_4_–C_4_ derivatives was divided into two steps. First, 2 g of semi-refined alginate containing 810 mg of saccharidic materials was dispersed in water (60 mL) under reflux in the presence of acid (MSA, 5 eq) to reduce the degree of polymerization. After 8 h reflux, butanol (60 mL) was added, and the Dean-Stark set-up was used to allow the removal of water by azeotropic distillation (15 h). The overall reaction time (23 h) and the quantity of MSA (5 eq) were increased to optimize the conversion rate (Scheme 6). Then, transglycosylation and transesterification reactions with dodecanol were carried out in the same pot. The influence of the MSA amount added in the second step was studied. It was found that a better yield was obtained when no additional acid was added. Indeed, with more MSA, there were more degradation products to be formed. As for the oligosaccharides, the one-pot saponification step was achieved with the mixture resulting from the second step. The concentration of the NaOH solution used was decreased (0.2 N NaOH, 3 eq, 70 °C, 1 h) in order to have a larger quantity in the aqueous phase and thus a better dispersion of the organic phase. Water was then eliminated by freeze-drying. Solid-liquid extraction with EtOAc allowing the removal of dodecanol was evaluated from this raw material. Unfortunately, a loss of *n*-dodecyl α,β d-glucopyranosides **16** and *n*-dodecyl fucosides derivatives **17**,**18** was observed. As these compounds are not in salt form like the uronate derivatives, they were solubilized by EtOAc and carried along with the dodecanol in the filtrate. In order to limit the loss of these products of interest, other solvents were tested: isopropanol, anisole, acetone, methyl isobutyl ketone, methyl ethyl ketone, 2-methyltetrahydrofuran, heptane, cyclohexanone, and acetonitrile. The only solvents that did not allow the non-ionic derivatives to be lost completely were acetone and acetonitrile. The best results were obtained with the latter. However, in the interest of environmental compatibility, acetone was finally chosen. The isolated mixture contains 98.5% dry matter and 40.7% mineral matter. At this stage, due to the difficulty of potential scale-up and the risk of emulsion formation, an alternative method for the final purification was developed. The mixture obtained after solid-liquid extraction with acetone was first dissolved in ice-cold water and then acidified with an oxalic acid solution to a pH of about 2. After concentration by freeze-drying, the mixture was taken up in acetone. The products were solubilized while the salts precipitated. After filtration, the products were recovered in the filtrate. Oxalic acid was chosen for its eco-compatibility and its pKa_1_ of 1.25, which allows the sodium carboxylate function of the uronate derivatives (pKa ≈ 4) to be protonated without the risk of also protonating the sodium methane sulfonate (pKa = −1.92). It is also to avoid finding methane sulfonic acid in the filtrate that the pH of the aqueous solution should not be decreased below 2. This cascading one-pot method with a final purification step is applicable on an industrial scale and it allows the elimination of fatty alcohols in addition to salts. ^1^H NMR analysis of the **H**–**C_12_ s-r Alg** composition revealed the presence of the following products (Table 9): *n*-dodecyl α-d-mannopyranosiduronic acid **13α** (23 mol%), *n*-dodecyl α-d-mannofuranosidurono-6,3-lactone **9α** (5 mol%), *n*-dodecyl β-d-mannofuranosidurono-6,3-lactone **9β** (13 mol%), *n*-dodecyl β-l-gulopyranosiduronic acid **14β** (11 mol%**)**, *n*-dodecyl β-l-gulofuranosidurono-6,3-lactone **10β** (20 mol%), *n*-dodecyl-α-l-fucopyranosides **17α** (5 mol%), *n*-dodecyl-β-l-fucopyranosides **17β** (8 mol%), and *n*-dodecyl-α,β-l-fucofuranosides **18α,β** (15 mol%) (Figures S15 and S16, Supplementary Materials). ^1^H NMR analysis of the **C_12_ s-r Alg** composition did not allow the identification of signals corresponding to *n*-dodecyl α,β d-glucopyranosides **16**. Furthermore, a significant amount of *n*-dodecyl α,β d-glucopyranosides **16** and *n*-dodecyl fucosides derivatives **17**,**18** was solubilized in acetone which means that the quantity of alkyl glycosides present in the final surfactant compositions has decreased significantly following the purification steps.

### 2.4. Synthesis from Crude Brown Seaweeds

The final challenge was to extend the cascading one-pot synthesis technology developed from oligo- and polysaccharide alginates to commercial milled brown seaweeds rich in alginate and fucoidan polysaccharides (Asco T10 from the *Ascophyllum nodosum* species, Thorverk, Iceland). Indeed, the use of such crude seaweeds could contribute to lowering the production cost of surfactant compositions due to the reduced price of these raw materials compared to refined or semi-refined polysaccharides and to a simplification of the process which no longer requires the prior extraction of alginates.

**Scheme 7:**
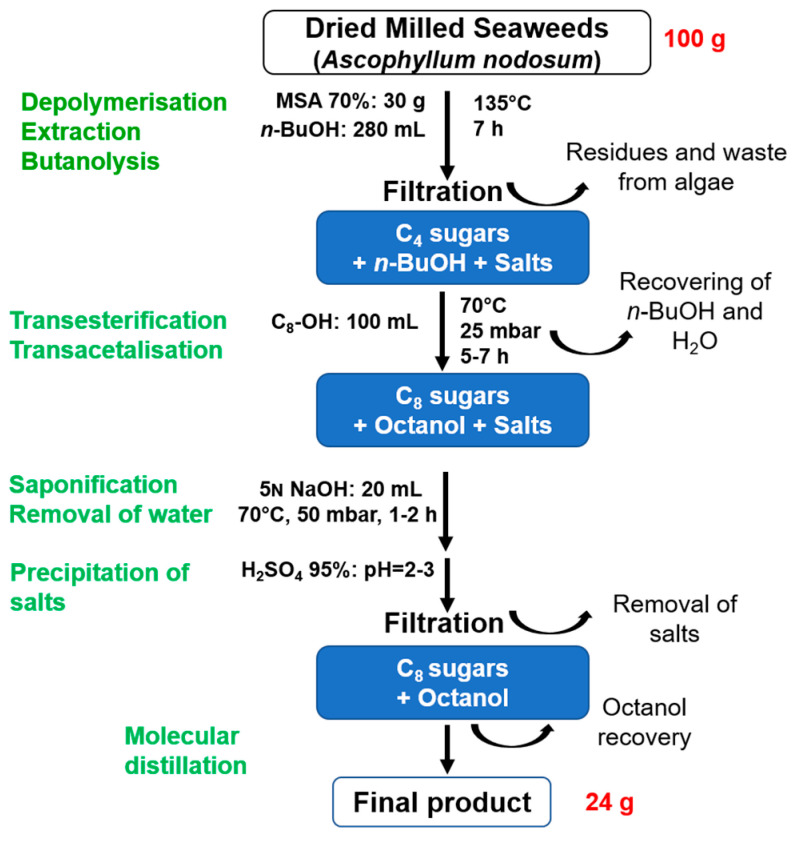
Cascading one-pot process for the production of surfactant composition from crude seaweeds.

The process was developed on a 100 g scale of crude seaweeds in a 1 L reactor. It was shown that it was possible to combine the depolymerization, extraction, and butanolysis steps simultaneously by immersing the milled algae in the butanol solution in the presence of the acid catalyst [20]. In this case, it was observed that the addition of water was not necessary and that the residual moisture of the algae (13 wt%) was sufficient to carry out the depolymerization. In practice, the algae behave like sponges by absorbing the butanol. This optimization made it possible to reduce the amount of butanol, to avoid adding water, and to dispense with the Dean-Stark apparatus that was previously required. A standard distillation set-up was used, which greatly simplified the scaling up of the process. Thus, milled seaweeds (100 g) were stirred in butanol (280 mL) at 135 °C for 7 h in the presence of MSA 70% (30 g). The resulting mixture composed of dibutylated uronates (alginate) and butylated fucosides (fucoidan) was then filtered on sintered glass to eliminate the insoluble materials such as salts or the fibers contained in the seaweeds (Scheme 7).

The second step of the process based on transesterification/transglycosylation reactions led to the replacement of butyl chains by longer chains using a fatty alcohol as well as the recycling of butanol through a vacuum distillation. Octanol was selected as the fatty alcohol to obtain C_8_-surfactants. The advantage of this alcohol was its liquid state at room temperature which facilitated its manipulation during the process, and also its capacity to be eliminated by distillation. The C_8_-C_8_ derivatives were then obtained after stirring for 7 h at 70 °C under reduced pressure. The anionic charge of the uronate derivatives was recovered after a saponification step with a 5N aqueous solution of NaOH for 1 h at 65 °C. The salts and the excess alcohol were then removed from the final mixture. For that purpose, residual water was first eliminated by evaporation under reduced pressure, followed by acidification of the medium through the addition of concentrated sulfuric acid to a pH value of about 2–3. This acidification step solubilized the uronate and fucoside derivatives in the residual alcohol and precipitated the salts. Thus, the salts were easily removed by filtration in addition to the other insoluble elements in octanol. Finally, the oily filtrate was treated by molecular distillation using a thin-film evaporator under high vacuum (2 mbar) at a temperature of around 90 °C. Using this technology, octanol was recovered in a very efficient way while minimizing the risk of degradation of the sugar derivatives present in the final mixture.

**Figure 2:**
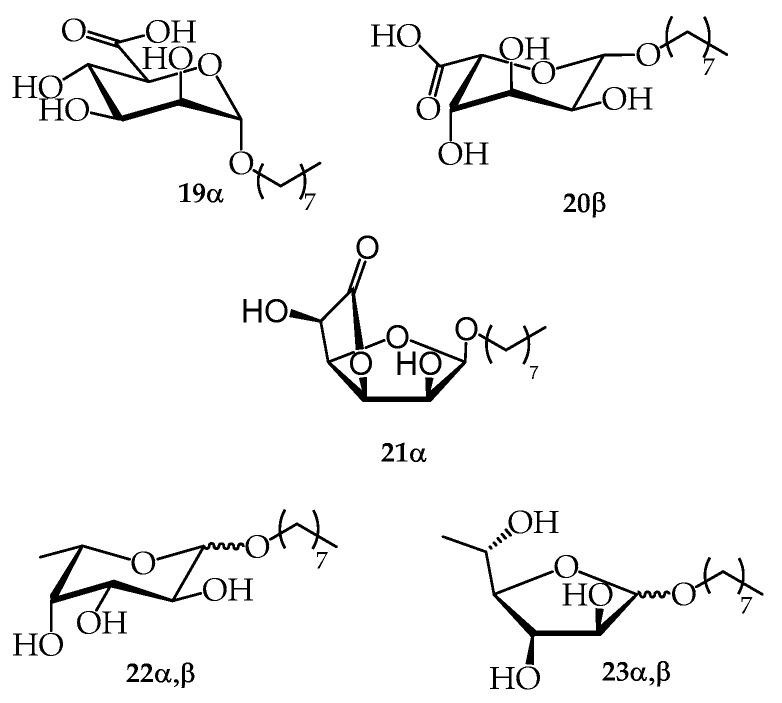
Proposed surfactant composition **H**–**C_8_ crude Alg** based on ^1^H NMR analysis.

This overall process allowed the production of 24 g of a surfactant composition through a solvent-free process that included purification steps limited to acid/base reactions and filtration/distillation steps (Scheme 7). The isolated mixture was subjected to thermogravimetric analysis which revealed the presence of 83.2% organic matter, 10.5% water, and 6.75% ash. A ^1^H NMR analysis in CD_3_OD was also performed, based on the integration of the anomeric sugar proton signals in the 4.2–5.0 ppm region (Table 10), which showed that the surfactant composition (Figure 2) contained 30 mol% *n*-octyl α-d-mannopyranosiduronic acid **19α**, 6 mol% *n*-octyl β-l-gulopyranosiduronic acid **20β**, 5% *n*-octyl α-l-gulofuranosidurono-6,3-lactone **21α**, 13 mol% *n*-octyl-α-l-fucopyranoside **22α**, 9 mol% *n*-octyl-β-l-fucopyranoside **22β**, 9 mol% *n*-octyl-α-l-fucofuranoside **23α**, and 28 mol% *n*-octyl-β-l-fucofuranoside **23β** (Figures S17 and S18, Supplementary Materials). The integration of the doublet relative to the fucosyl methyl group at 1.19–1.27 ppm confirmed this percentage of fucosides in the final mixture. ^1^H NMR study of the surfactant composition did not allow us to identify signals corresponding to *n*-octyl α,β d-glucopyranosides. The surface tension measurements obtained with this H–C_8_ surfactant composition are presented in Section 2.5.

### 2.5. Physico-Chemical Properties of Anionic and Non-Ionic Surfactant Compositions Derived from Oligoalginates, Semi-Refined Alginates, and Crude Brown Seaweed

Surface activities at the air-water interface were investigated for compositions **H**–**C_12_ Man**, **H**–**C_12_ Gul**, **H**–**C_12_ OAlg**, and **H**–**C_12_ s-r Alg** in addition to the composition obtained from the crude seaweeds. Furthermore, biodegradability and aquatic ecotoxicity were evaluated for the **H**–**C_12_ s-r Alg** composition.

#### 2.5.1. Measurements of Air-Water Interfacial Behavior

##### H-C_12_ Derivatives from OM, OG, OAlg, and s-r Alg

**Figure 3:**
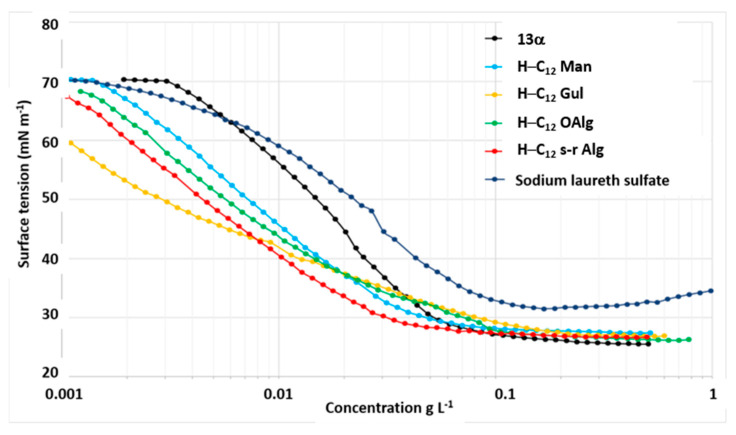
Values of the surface tension of H–C_12_-based surfactant compositions derived from dodecanol.

The different batches of H-C_12_ were characterized by surface tension measurements (Figure 3). The results show that all surfactant compositions are effective in reducing the surface tension of the water, since values below 30 mN m^−1^ were measured for the CMCs. Pure *n*-dodecyl α-d-mannopyranosiduronic acid **13α** [16] exhibits a CMC of 0.06 g L^−1^ and a γ_CMC_ of 28.9 mN m^−1^. Among the different surfactant compositions, it lowered the surface tension of water the least in the low concentration values. The **H**–**C_12_ Man** composition gives the same CMC and γ_CMC_ values (0.06 g L^−1^ and 29.1 mN m^−1^), but it lowers the surface tension much more effectively at low concentration values. This indicates, therefore, that the presence of **H**–**C_12_ Man** isomers allows for improved surfactant performance. The mixture from oligoguluronate gives the highest CMC with a value of 0.12 g L^−1^ and a γ_CMC_ of 28.8 mN m^−1^. However, for concentrations below 0.008 g L^−1^, the lowest surface tension values were measured with this **H**–**C_12_ Gul** composition. Similar CMC and γ_CMC_ values were obtained with the batch from oligoalginate (0.11 g L^−1^, 27.6 mN m^−1^). Finally, the surfactant mixture obtained from semi-refined alginate provided the lowest CMC with a value of 0.04 g L^−1^ and a γ_CMC_ of 29.0 mN m^−1^. Tensiometry measurements were also carried out on anionic surfactant, sodium laureth sulfate (**SLES**). The CMC was determined at 0.13 g L^−1^ and the γ_CMC_ at 31.9 mN m^−1^. Thus, all the compounds obtained from the different sources of algae extracts exhibit better performance in terms of CMC and γ_CMC_.

##### H–C_8_ Surfactant Composition from Crude Seaweed

Surprisingly, surface tension measurements in pH 7 buffered solution from the composition derived from crude seaweed and octanol (**H**–**C_8_ crude seaweed**) revealed a CMC value of 0.007 g L^−1^ and a γ_CMC_ of 27.0 mN m^−1^, demonstrating excellent surfactant properties.

#### 2.5.2. Ecotoxicity Studies

A series of ecotoxicity studies have been performed with the uronic surfactant compositions derived from semi-refined alginate and dodecanol (**H**–**C_12_ s-r Alg**) in addition to standard **SLES**. Experiments to determine the ecotoxicity of products include effects on aquatic organisms, from microalgae to fish. These different pollutant-sensitive organisms serve as controls. Three standardized tests were conducted: an algal growth inhibition test, a microcrustaceous immobilization test, and a lethal toxicity test on freshwater fish. The microalgae used, called *Pseudokirchneriella subcapitata*, are ubiquitous in the environment and are sensitive to toxic substances. The test is conducted according to the OECD 201 method [21], which corresponds to the percentage of inhibition of the algal growth rate after a 72-h incubation period (CEr50). The microcrustaceans used are Daphnia Magna. They are freshwater crustaceans that are an important nutritional source for many aquatic organisms and their presence in sufficient numbers and in good health helps to maintain a certain balance in their ecosystem. The Daphnia Magna have the distinction of being extremely susceptible to changes, even minor ones, in the composition of their aquatic environment. The OECD 202 test [22] is based on the determination of the CE50 concentration which, in 24 h and/or 48 h, immobilizes 50% of the microcrustaceans experimented on. Finally, the test on freshwater fish is conducted according to the OECD 203 method [23] to determine the concentration for which the sample has lethal toxicity for 50% of a *Brachydanio rerio* test population after a 96-h exposure period (CL50). *Brachydanio rerio* is one of the model organisms commonly encountered in research laboratories for fish behavior studies [24]. These tests are based on acute aquatic toxicity tests, i.e., adverse effects on aquatic organisms during short-term exposure. The results observed based on the concentration of the samples to be analyzed allow the substances to be categorized into different categories, as shown in Table 11.

As shown in Table 12, **SLES** can be considered toxic to algae and is borderline toxic to Daphnia and fish. In comparison, **H**–**C_12_ s-r Alg** has a reduced ecotoxicity since it is not very toxic to algae and fish and not toxic to Daphnia.

#### 2.5.3. Biodegradability Studies

The comparative aerobic readily biodegradability of compounds **H–C_12_ s-r Alg** and **SLES** was finally studied using the OECD 301 B method [25]. The objective of this test is to determine the release of carbon dioxide by microbial digestion in the aerobic environment of the compound to be analyzed. During the test, the compound is placed in a watery medium to which is added a mixed seeding of a plant dealing with urban wastewater. The surfactant studied is the only source of carbon and energy and it is introduced at a theoretical concentration of 10 mg L^−1^ of dissolved organic carbon (COD). The CO_2_ formed during degradation is trapped in external containers. The tests are conducted over 28 days, during which the evolution of the biodegradation rate is determined. The OECD 301 B method considers a product to be readily biodegradable if the biodegradation rate has reached at least 60% after 28 days and readily biodegradable with respect to the 10-day period if the biodegradation rate has reached at least 60% rate 10 days after the rate has reached 10%.

As the limit of 60% biodegradation was reached, both products are readily biodegradable but **H**–**C_12_ s-r Alg**_,_ exhibited lower biodegradability rates after 28 days (72%) than **SLES** (94%). Under the experimental conditions of the test, **H**–**C_12_ s-r Alg**_,_ (Figure S19, Supplementary Materials) is considered to be readily biodegradable without meeting the 10-day interval. Indeed, the CO_2_ release threshold of 60% of theoretical CO_2_ was not reached within the 10-day interval, but within the first 28 days of testing (threshold reached on day 21). In contrast, for **SLES**, the CO_2_ release threshold of 60% of the theoretical CO_2_ was reached within 10 days (Figure S20, Supplementary Materials).

## 3. Discussion

To facilitate the industrial development of innovative surfactants based on carboxylate saccharides, we have proposed a new approach to produce these surfactants without sulfate and without ethylene oxide, according to a smart strategy based on a multi-step process carried out in a cascading single-pot mode without separating and isolating any reaction intermediates and applicable to oligomeric or polymeric alginates in a more or less refined form. The innovation of this research is also based on its positioning in green/blue chemistry (solvent-free reactions with little waste, biodegradable reagents, valorization of marine plant biomass, and eco-compatible products). The syntheses involve a succession of chemical reactions such as depolymerization by acid hydrolysis of oligo- or polysaccharides, esterification and glycosylation with *n*-butanol, transesterification and transglycosylation with fatty alcohols, and saponification. A final treatment involving mainly solid-liquid extraction, acidification, precipitation in an organic solvent (EtOAc or acetone), and filtration steps allows the final compositions to be enriched with active ingredients by removing (at least partially) residual salts and fatty alcohols. In terms of performance, the carboxylic acid-based surfactant compositions allow surface tension to be lowered to values ≤30 mN m^−1^ at concentrations equivalent to or lower than that of sodium laureth sulfate. In particular, the compositions derived from semi-refined alginate (s-r Alg) or oligomannuronate (OM) lead to CMC values two to three times lower than that obtained from sodium laureth sulfate. In addition, a significant improvement in ecotoxicity profile was observed for the biodegradable surfactant composition **H**–**C_12_ s-r Alg**, especially towards Daphnia.

In order to further simplify the process and reduce costs, a novel strategy has been successfully tested based on the direct use of plant materials in their raw state, i.e., without any chemical transformation, in particular solvent extraction or enzymatic extraction, and at most a mechanical and/or physical transformation, such as washing, grinding, and/or drying. A notable advantage of this approach is the use of plant materials with a water content of up to 13% by weight, thus avoiding the addition of water during the depolymerization step by hydrolysis of the polysaccharides. The process has been applied to brown seaweed containing alginates and fucans and/or fucoidans. The process incorporates the same reaction sequences as for polysaccharides (acid hydrolysis, esterification/glycosylation, transesterification/transglycosylation, and saponification) with the addition of a preliminary extraction step carried out in situ as well as filtration, centrifugation, or distillation steps under reduced pressure, allowing the elimination of the residues of plant material not involved in the syntheses and the recycling of the excess alcohols. The obtained composition **H**–**C_8_ crude Alg** is rich in carboxylic acid sugars, which also contain alkyl glycosides derived from neutral sugars representative of the structure of the polysaccharides present in the starting plant material (d-fucose). These modify the physicochemical properties of the final compositions and optimise their surface-active performance [20]. Indeed, low values of surface tension (27.0 mN m^−1^) and critical micellar concentration (0.007 g L^−1^) were obtained in the case of these mixtures of uronic acid- and fucose-based surfactants.

## 4. Materials and Methods

### 4.1. Chemistry

Oligomannnuronate, oligoguluronate, oligoalginate, and semi-refined alginate were produced from fresh or dry seaweeds (CEVA, 83 Rue de Pen Lan, 22610 Pleubian, France) [16,17,18]. Dried milled brown seaweeds (Asco T10) were purchased from Thorverk (Iceland) and all other commercially available chemicals were used without further purification. All reactions were monitored by thin-layer chromatography (Kieselgel 60F_254_ Merck). Compounds were visualized using a H_2_SO_4_ solution (5% H_2_SO_4_ in EtOH) or a vanillin solution (15 g of vanillin in 250 mL of EtOH and 2.5 mL of conc. H_2_SO_4_) followed by heating. Geduran 60 (40–63 µm, Merck) was used for column chromatography. NMR spectra were recorded on a Bruker Avance III 400 spectrometer operating at 400.13 MHz for ^1^H, equipped with a BBFO probe with a Z-gradient coil and a GREAT 1/10 gradient unit. The standard temperature was adjusted to 298 K. The *zg30* Bruker pulse program was used for 1D ^1^H NMR, with a TD of 64k, a relaxation delay d1 = 2 s, and 8 scans. The spectrum width was set to 18 ppm. Fourier transform of the acquired FID was performed with an apodization of 0.3 Hz in most of the cases. Chemical shifts are mentioned in parts per million (ppm) with tetramethylsilane as an internal standard. Coupling constants were expressed in Hertz (Hz) and the following abbreviations were used to indicate the multiplicity: s (singulet), d (doublet), t (triplet), q (quadruplet), m (multiplet), dd (doublet of doublets), dt (doublet of triplets), and br (broad signal).

Preparation of **C_4_**–**C_4_ Man** from OM

Oligomannuronate (500 mg, 1.81 mmol CO_2^−^_, 1 eq.) was dispersed in water (0.87 mL) and *n*-butanol (25 mL) in a round-bottom flask with a Dean-Stark apparatus. Technical-grade methanesulfonic acid 70% wt (422 mg, 3.08 mmol, 1.7 eq.) was added and the mixture was refluxed under vigorous stirring. The water formed in the medium was gradually removed by azeotropic distillation. After 7 h, the mixture was cooled to ambient temperature. The mixture was then neutralized with 1N NaOH solution (400 µL) and concentrated. The resulting mixture was dissolved in diethyl ether (20 mL) and then filtered using celite. The celite was finally rinsed with diethyl ether (100 mL) and the filtrate was concentrated. The products obtained (495 mg) were purified by silica gel chromatography with CH_2_Cl_2_:CH_3_OH, (97/3–96/4, *v*/*v*) to help determine the molar composition. The molar composition of this mixture of products before silica gel chromatography is as follows: 52% (*n*-butyl) *n*-butyl-α-d-mannopyranosiduronate (**1α**), 7% (*n*-butyl) *n*-butyl-α-d-mannofuranosiduronate (**2α**), 11% *n*-butyl-α-d-mannofuranosidurono-6.3-lactone (**3α**), 13% *n*-butyl-β-d-mannofuranosidurono-6.3-lactone (**3β**), 5% BDMF **(6)**, 5% *n*-butyl-α-l-gulofuranosidurono-6.3-lactone (**4α**), 5% *n*-butyl-β-l-gulofuranosidurono-6.3-lactone (**4β**), 3% (*n*-butyl) *n*-butyl-β-l-gulofuranosiduronate (**5β**). ^1^H NMR (400 MHz, CDCl_3_) δ 7.11 (d, *J* = 3.4 Hz, **BDMF**), 6.51 (dd, *J* = 3.5, 0.8 Hz, **BDMF**), 5.53 (s, **BDMF**), 5.14 (dd, *J* = 7.4, 4.7 Hz, **H3-4β**), 5.06 (s, **H1-4β**), 5.05 (d, *J* = 2.0 Hz, **H1-3α**), 5.03 (d, *J* = 4.6 Hz, **H1-3β**), 5.00 (t, *J* = 4.8 Hz, **H3-3α**), 4.95 (t, *J* = 4.5 Hz, **H1-4α**), 4.91 (d, *J* = 1.7 Hz, **H1-1α**), 4.90 (t, *J* = 5.0 Hz, **H3-3β**), 4.79 (dd, *J* = 6.8, 4.8 Hz, **H4-3β**), 4.76 (dd, *J* = 6.0, 4.5 Hz, **H4-3α**), 4.72 (dd, *J* = 7.6, 4.0 Hz, **H4-4β**), 4.69 (d, *J* = 5.5 Hz, **H4-4α**), 4.43 (d, *J* = 2.9 Hz, **H5-2α**),4.31–4.15 (m, OCH_2_), 4.08 (d, *J* = 9.5 Hz, **H5-1α**), 4.00 (t, *J* = 9.3 Hz, **H4-1α**), 3.94 (m, **H2-1α**), 3.87 (dd, *J* = 8.9, 3.5 Hz, **H3-1α**), 3.83–3.69 (m, OCH_2_), 3.65 (t, *J* = 6.6 Hz, **BDMF**), 3.60–3.38 (m, OCH_2_) 1.76–1.62 (m, _CH2_), 1.62–1.49 (m, CH_2_), 1.46–1.29 (m, CH_2_), 0.99–0.88 (m, CH_3_). After column chromatography, five fractions **F1**–**F5** were isolated and characterised by ^1^H NMR. **F1**: 57 mg of BDMF (**6**). (**Rf** = 0.94, CH_2_Cl_2_/MeOH (95/5, *v*/*v*)): ^1^H NMR (400 MHz, CDCl_3_) δ 7.11 (d, *J* = 3.4 Hz), 6.51 (dd, *J* = 3.5, 0.8 Hz), 5.53 (s), 4.36 (t, *J* = 6.7 Hz), 3.65 (t, *J* = 6.6 Hz), 1.80–1.63 (m, CH_2_), 1.63–1.50 (m, CH_2_), 1.48–1.31 (m, CH_2_), 1.00–0.86 (m, CH_3_); **F2**: 12 mg of *n*-butyl-α-l-gulofuranosidurono-6.3-lactone (**4α**) and (*n*-butyl) *n*-butyl-β-l-gulofuranosiduronate (**5β**). (**Rf** = 0.42, CH_2_Cl_2_/MeOH (95/5, *v*/*v*)): ^1^H NMR (400 MHz, CDCl_3_) δ 4.98 (t, *J* = 5.6 Hz, **H3-4α**), 4.95 (d, *J* = 4.5 Hz, **H1-4α**), 4.91 (s, **H1-5β**), 4.69 (dd, *J* = 5.5, 0.8 Hz, **H4-4α**), 4.52 (dd, *J* = 8.0, 1.3 Hz, **H4-5β**), 4.38 (d, *J* = 1.3 Hz, **H5-5β**), 4.28 (d, *J* = 0.7 Hz, **H5-4α**), 4.26–4.18 (m, OCH_2_), 4.15 (t, *J* = 5.1 Hz, **H2-4α**), 3.91 (d, *J* = 5.4 Hz, **H2-5β**), 3.71 (dt, *J* = 9.6, 6.7 Hz, OCH_2_), 3.58 (dt, *J* = 9.7, 6.7 Hz, OCH_2_), 3.43 (dt, *J* = 9.6, 6.7 Hz, OCH_2_), 1.73–1.59 (m, CH_2_), 1.58–1.47 (m, CH_2_), 1.46–1.24 (m, CH_2_), 0.99–0.86 (m, CH_3_); **F3**: 65 mg of *n*-butyl-β-d-mannofuranosidurono-6.3-lactone (**3β**), (*n*-butyl) *n*-butyl-α-d-mannofuranosiduronate (**2α**), *n*-butyl-α-l-gulofuranosidurono-6.3-lactone (**4α**), *n*-butyl-β-l-gulofuranosidurono-6.3-lactone (**4β**) and (*n*-butyl) *n*-butyl-β-l-gulofuranosiduronate (**5β**). (**Rf** = 0.38, CH_2_Cl_2_/MeOH (95/5, *v*/*v*)): ^1^H NMR (400 MHz, CDCl_3_) δ 5.14 (dd, *J* = 7.4, 4.7 Hz, **H3-4β**), 5.06 (s, **H1-4β**), 5.03 (d, *J* = 4.6 Hz, **H1-3β**), 4.97 (s, **H1-2α**), 4.95 (d, *J* = 4.5 Hz, **H1-4α**), 4.90 (t, *J* = 5.0 Hz, **H3-3β**), 4.79 (dd, *J* = 6.8, 4.8 Hz, **H4-3β**), 4.72 (dd, *J* = 7.6, 4.0 Hz, **H4-4β**), 4.69 (d, *J* = 5.5 Hz, **H4-4α**), 4.56 (d, *J* = 4.0 Hz, **H5-4β**), 4.52 (d, *J* = 8.0 Hz, **H4-5β**), 4.43 (d, *J* = 2.9 Hz, **H5-2α**), 4.39 (d, *J* = 7.4 Hz, **H5-3β**), 4.30–4.19 (m, OCH_2_), 3.93 (d, *J* = 4.7 Hz, **H2-2α**), 3.79 (dt, *J* = 9.5, 6.7 Hz, OCH_2_), 3.66 (dt, *J* = 9.6, 6.7 Hz, OCH_2_), 3.54–3.38 (m, OCH_2_), 1.73–1.47 (m, CH_2_), 1.45–1.24 (m, CH_2_), 0.93 (m, CH_3_); **F4**: 49 mg of *n*-butyl-β-d-mannofuranosidurono-6.3-lactone (**3β**), *n*-butyl-α-d-mannofuranosidurono-6.3-lactone (**3α**) and *n*-butyl-β-l-gulofuranosidurono-6.3-lactone(**4β**). (**Rf** = 0.29, CH_2_Cl_2_/MeOH (95/5, *v*/*v*)): ^1^H NMR (400 MHz, CDCl_3_) δ 5.13 (dd, *J* = 7.4, 4.7 Hz, **H3-4β**), 5.05 (d, *J* = 2.0 Hz, **H1-3α**), 5.03 (d, *J* = 4.6 Hz, **H1-3β**), 5.00 (t, *J* = 4.8 Hz, **H3-3α**), 4.90 (t, *J* = 4.8 Hz, **H3-3β**), 4.79 (dd, *J* = 6.8, 4.8 Hz, **H4-3β**), 4.76 (dd, *J* = 6.0, 4.5 Hz, **H4-3α**), 4.72 (dd, *J* = 7.6, 4.0 Hz, **H4-4β**), 4.27–4.17 (m, OCH_2_), 3.74–3.61 (m, OCH_2_), 3.50–3.35 (m, OCH_2_), 1.70–1.46 (m, CH_2_), 1.42–1.27 (m, CH_2_), 0.96–0.86 (m, CH_3_); **F5**: 183 mg of (*n*-butyl) *n*-butyl-α-d-mannopyranosiduronate (**1α**). (**Rf** = 0.22, CH_2_Cl_2_/MeOH (95/5, *v*/*v*)): ^1^H NMR (400 MHz, CDCl_3_) δ 4.90 (d, *J* = 1.7 Hz, **H1-1α**), 4.28–4.15 (m, OC**H_2_**), 4.28–4.15 (m, OCH**_2_**), 4.08 (d, *J* = 9.5 Hz, **H5-1α**), 4.00 (t, *J* = 9.3 Hz, **H4-1α**), 3.93 (dd, *J* = 3.4, 1.8 Hz, **H2-1α**), 3.86 (dd, *J* = 9.0, 3.4 Hz, **H3-1α**), 3.72 (dt, *J* = 9.7, 6.7 Hz, OCH_2_), 3.50–3.43 (m, OCH_2_), 1.72–1.62 (m, CH_2_), 1.62–1.51 (m, CH_2_), 1.44–1.30 (m, CH_2_), 0.93 (m, CH_3_).

Preparation of **C_12_**–**C_12_ Man** from OM

Oligomannuronate (500 mg, 1.81 mmol CO_2_^-^, 1 eq.) was dispersed in water (0.9 mL) and *n*-butanol (25 mL) in a round-bottom flask with a Dean-Stark apparatus. Methanesulfonic acid technical grade 70% wt (419 mg, 3.05 mmol, 1.7 eq.) was added and the mixture was refluxed under vigorous stirring. The water formed in the medium was gradually removed by azeotropic distillation. After 7 h, the mixture was cooled to ambient temperature. Dodecanol (1.6 mL, 7.2 mmol, 4 eq.) and the 70% methanesulfonic acid solution (246 mg, 1.8 mmol, 1 eq.) were added. The mixture was stirred at 70 °C under reduced pressure (up to 5 mbar) using distillation apparatus. Once the butanol had been completely removed (2.17 h), water (16 mL) was added and the mixture was then neutralized with 1N NaOH solution (0.5 mL). The whole mixture was left to stir vigorously at 80 °C for 1 h and the mixture was cooled to ambient temperature. The mixture was cooled at 0 °C to solidify the organic phase which was isolated by solid-liquid extraction and dried under vacuum. The resulting mixture (1.684 g) was purified by a first silica gel chromatography with CH_2_Cl_2_/CH_3_OH, (100:0–96:4) to give crude mixture **C_12_**–**C_12_ Man** (504 mg). The molar composition of this mixture is as follows: 48% (*n*-dodecyl) *n*-dodecyl-α-d-mannopyranosiduronate (**7α**), 9% (*n*-dodecyl) *n*-dodecyl-α-d-mannofuranosiduronate (**8α**), 5% *n*-dodecyl-α-d-mannofuranosidurono-6.3-lactone (**9α**), 22% *n*-dodecyl-β-d-mannofuranosidurono-6.3-lactone (**9β**), 3% *n*-dodecyl-α-l-gulofuranosidurono-6.3-lactone (**10α**), 3% *n*-dodecyl-β-l-gulofuranosidurono-6.3-lactone (**10β**), 5% (*n*-dodecyl) *n*-dodecyl-β-l-gulopyranosiduronate (**11β**), 6% (*n*-dodecyl) *n*-dodecyl-β-l-gulofuranosiduronate (**12β**). ^1^H NMR (400 MHz, CDCl_3_) δ 5.07 (s, **H1-10β**), 5.05 (d, *J* = 2.0 Hz, **H1-9α**), 5.03 (d, *J* = 4.5 Hz, **H1-9β**), 4.99 (t, *J* = 4.7 Hz, **H3-9α**), 4.97 (t, *J* = 5.7 Hz, **H3-10α**), 4.97 (s, **H1-8α, H2-9α**), 4.95 (d, *J* = 4.5 Hz, **H1-10α**), 4.92 (d, *J* = 1.7 Hz, **H1-7α**), 4.91 (s, **H1-12β**), 4.90 (t, *J* = 5.0 Hz, **H3-9β**), 4.79 (dd, *J* = 6.8, 4.8 Hz, **H4-9β**), 4.72 (dd, *J* = 7.4, 4.0 Hz, **H4-10β**), 4.69 (d, *J* = 5.6 Hz, **H4-10α**), 4.58 (d, *J* = 4.0 Hz, **H5-10β**), 4.55 (d, *J* = 1.0 Hz, **H5-11β**), 4.53 (dd, *J* = 7.9, 1.4 Hz, **H4-12β**), 4.43 (d, *J* = 2.9 Hz, **H5-8α**), 4.39 (d, *J* = 7.0 Hz, **H5-9β**), 4.29–4.14 (m, OCH_2_), 4.09 (d, *J* = 9.6 Hz, **H5-7α**), 4.00 (t, *J* = 9.2 Hz, **H4-7α**), 3.95 (dd, *J* = 3.4, 1.7 Hz, **H2-7α**), 3.89 (dd, *J* = 8.9, 3.4 Hz, **H3-7α**), 3.82–3.68 (m, OCH_2_), 3.53–3.37 (m, OCH_2_), 1.74–1.64 (m, CH_2_), 1.63–1.53 (m, CH_2_), 1.38–1.20 (m, CH_2_), 0.91–0.85 (m, CH_3_). A second silica gel chromatography with CH_2_Cl_2_/CH_3_OH, (97:3) was made to help determine the molar composition. Five fractions **F1**–**F5** were isolated and characterised by ^1^H NMR. **F1**: 74 mg of (*n*-dodecyl) *n*-dodecyl-α-d-mannofuranosiduronate (**8α**), *n*-dodecyl-β-d-mannofuranosidurono-6.3-lactone (**9β**), *n*-dodecyl-α-l-gulofuranosidurono-6.3-lactone (**10α**) and (*n*-dodecyl) *n*-dodecyl-β-l-gulopyranosiduronate (**12β**). (**Rf** = 0.47, CH_2_Cl_2_/MeOH (95/5, *v*/*v*)): ^1^H NMR (400 MHz, CDCl_3_) δ 5.03 (d, *J* = 4.5 Hz, **H1-9β**), 4.98 (t, *J* = 5.8 Hz, **H3-10α**), 4.97 (s, **H1-8α**), 4.96 (d, *J* = 4.5 Hz, **H1-10α**), 4.91 (s, **H1-12β**), 4.89 (t, *J* = 5.0 Hz, **H3-9β**), 4.79 (dd, *J* = 6.8, 4.8 Hz, **H4-9β**), 4.70 (dd, *J* = 5.7, 0.8 Hz, **H4-10α**), 4.71–4.65 (m, **H3-12β**), 4.65–4.61 (m, **H3-H4-8α**), 4.52 (dd, *J* = 7.9, 1.3 Hz, **H4-12β**), 4.43 (d, *J* = 2.8 Hz, **H5-8α**), 4.37 (d, *J* = 1.3 Hz, **H5-12β**), 4.27–4.18 (m, OCH_2_), 3.92 (d, *J* = 4.5 Hz, **H2-8α**), 3.81–3.54 (m, OCH_2_), 1.76–1.63 (m, CH_2_), 1.62–1.48 (m, CH_2_), 1.39–1.20 (m, CH_2_), 0.91–0.85 (m, CH_3_); **F2:** 64 mg of *n*-dodecyl-α-d-mannofuranosiduronate (**8α**), *n*-dodecyl-β-d-mannofuranosidurono-6.3-lactone (**9β**). (**Rf** = 0.45, CH_2_Cl_2_/MeOH (95/5, *v*/*v*)): ^1^H NMR (400 MHz, CDCl_3_) δ 5.03 (d, *J* = 4.6 Hz, **H1-9β**), 4.97 (s, **H1-8α**), 4.90 (t, *J* = 5.0 Hz, **H3-9β**), 4.79 (dd, *J* = 6.8, 4.8 Hz, **H4-9β**), 4.62 (m, **H3-H4-8α**), 4.42 (d, *J* = 2.9 Hz, **H5-8α**), 4.38 (d, *J* = 6.6 Hz, **H5-9β**), 4.29–4.18 (m, OCH2), 3.92 (d, *J* = 4.7 Hz, **H2-8α**), 3.78 (dt, *J* = 9.5, 6.8 Hz, OCH_2_), 3.65 (dt, *J* = 9.5, 7.3 Hz, OCH_2_), 3.53–3.35 (m, OCH_2_)), 1.74–1.63 (m, CH_2_), 1.63–1.50 (m, CH_2_), 1.38–1.19 (m, CH_2_), 0.91–0.85 (m, CH_3_); **F3**: 30 mg of (*n*-dodecyl) *n*-dodecyl-α-d-mannopyranosiduronate (**7α**), (*n*-dodecyl) *n*-dodecyl-α-d-mannofuranosiduronate (**8α**), *n*-dodecyl-α-d-mannofuranosidurono-6.3-lactone (**9α**), *n*-dodecyl-β-d-mannofuranosidurono-6.3-lactone (**9β**), *n*-dodecyl-β-l-gulofuranosidurono-6.3-lactone (**10β**), (*n*-dodecyl) *n*-dodecyl-α-l-gulopyranosiduronate (**11α**), (*n*-dodecyl) *n*-dodecyl-β-l-gulopyranosiduronate (**11β**), and (*n*-dodecyl) *n*-dodecyl-β-l-gulofuranosiduronate (**12β**). (**Rf** = 0.38, CH_2_Cl_2_/MeOH (95/5, *v*/*v*)): ^1^H NMR (400 MHz, CDCl_3_) δ 5.14 (dd, *J* = 7.4, 4.7 Hz, **H3-10β**), 5.07 (s, **H1-10β**), 5.05 (d, *J* = 2.0 Hz, **H1-9α**), 5.03 (d, *J* = 4.5 Hz, **H1-9β**), 4.99 (t, *J* = 4.7 Hz, **H3-7α**), 4.97 (s, **H2-9α**), 4.92 (d, *J* = 1.7 Hz, **H5-7α**), 4.91 (s, **H1-12β**), 4.89 (t, *J* = 5.0 Hz, **H3-9β**), 4.78 (dt, *J* = 5.8, 4.6 Hz, **H4-9α,β**), 4.72 (dd, *J* = 7.5, 4.0 Hz, **H4-10β**), 4.65–4.61 (m, **H3-H4-8α**), 4.63 (d, *J* = 7.8 Hz, **H1-11β**), 4.60 (d, *J* = 1.7 Hz, **H5-11α**), 4.57 (d, *J* = 4.0 Hz, **H5-10β**), 4.55 (d, *J* = 1.5 Hz, **H5-11β**), 4.52 (dd, *J* = 7.9, 1.0 Hz, **H4-12β**), 4.43 (d, *J* = 2.9 Hz, **H5-8α**), 4.40 (d, *J* = 1.3 Hz, **H5-12β**), 4.39 (d, *J* = 6.3 Hz, **H5-9β**), 4.27–4.18 (m, OCH_2_), 4.13 (dd, *J* = 4.0, 1.7 Hz, **H4-11β**), 4.09 (d, *J* = 9.5 Hz, **H5-7α**), 3.80–3.64 (m, OCH_2_), 3.55–3.37 (m, OCH_2_), 1.73–1.48 (m, CH_2_), 1.42–1.18 (m, CH_2_), 0.93–0.84 (m, CH_3_); **F4:** 240 mg of (*n*-dodecyl) *n*-dodecyl-α-d-mannopyranosiduronate (**7α**). (**Rf** = 0.3, CH_2_Cl_2_/MeOH (95/5, *v*/*v*)): ^1^H NMR (400 MHz, CDCl_3_) δ 4.91 (d, *J* = 1.7 Hz, **H1-7α**), 4.21 (m, OCH_2_), 4.08 (d, *J* = 9.6 Hz, **H5-7α**), 4.01 (t, *J* = 9.3 Hz, **H4-7α**), 3.94 (dd, *J* = 3.4, 1.8 Hz, **H2-7α**), 3.88 (dd, *J* = 8.9, 3.4 Hz, **H3-7α**), 3.72 (dt, *J* = 9.7, 6.8 Hz, OCH_2_), 3.47 (dt, *J* = 9.7, 6.5 Hz, OCH_2_), 1.73–1.64 (m, CH_2_), 1.64–1.52 (m, CH_2_), 1.38–1.22 (m, CH_2_), 0.92–0.84 (m, CH_3_); **F5**: 9 mg of a mixture of products (numerous ^1^H NMR signals).

Preparation of **H**–**C_12_ Man** from OM

Oligomannuronate (500 mg, 1.81 mmol CO_2^−^_, 1 eq.) was dispersed in water (1 mL) and *n*-butanol (25 mL) in a round-bottom flask with a Dean-Stark apparatus. Methanesulfonic acid technical grade 70% wt (422 mg, 3.08 mmol, 1.7 eq.) was added and the mixture was refluxed under vigorous stirring. The water formed in the medium was gradually removed by azeotropic distillation. After 7 h, the mixture was cooled to ambient temperature. Dodecanol (1.6 mL, 7.2 mmol, 4 eq.) and the 70% methanesulfonic acid solution (248 mg, 1.81 mmol, 1 eq.) were added. The mixture was stirred at 70 °C under reduced pressure (up to 5 mbar) using distillation apparatus. Once the butanol had been completely removed (1.42 h), a 0.4N NaOH solution (13.5 mL, 5.4 mmol, 3.0 eq.) was added and the mixture was left to stir vigorously at 70 °C. for 1 h. The water was then removed by freeze-drying or by azeotropic distillation with butanol. The excess dodecanol present in the crude product was removed by solid-liquid extraction with EtOAc. At the end of this treatment, the mixture of products was dissolved in ice-cold water (15 mL) and EtOAc (22.5 mL) followed by a 1N hydrochloric acid solution (3.1 mL) was added. The aqueous solution was extracted with EtOAc (5 × 7.5 mL). The organic phases were combined and washed with a saturated NaC1 solution (30 mL) and a 1N hydrochloric acid solution (150 µL). The organic phase was dried with MgSO_4_ and then concentrated under vacuum. A mixture of products **H**–**C_12_ Man** was obtained (341 mg), the molar composition of which is: 47% *n*-dodecyl-α-d-mannopyranosiduronic (**13α**), 4% *n*-dodecyl-α-d-mannofuranosidurono-6.3-lactone (**9α**), 12% *n*-dodecyl-β-d-mannofuranosidurono-6.3-lactone (**9β**), 4% *n*-dodecyl-β-l-gulopyranosiduronic (**14β**), 9% *n*-dodecyl-α-l-gulofuranosidurono-6,3-lactone (**10α**), 9% *n*-dodecyl-β-l-gulofuranosidurono-6,3-lactone (**10β**), 4% *n*-dodecyl-β-l-gulofuranosiduronic acid (**15β**), 10% non-identified molecule **X**. ^1^H NMR (400 MHz, CD_3_OD) δ 5.11 (d, *J* = 6.5 Hz, **X**), 5.07 (dd, *J* = 7.6, 4.7 Hz, **H3**-**10β**), 5.00 (d, *J* = 1.6 Hz, **H1**-**9α**), 4.98 (s, **H1**-**10β**), 4.97 (t, *J* = 5.7 Hz, **H3-10α**), 4.96 (d, *J* = 3.8 Hz, **H1-9β**), 4.92 (d, *J* = 4.4 Hz, **H1-10α**), 4.80 (d, *J* = 2.1 Hz, **H1-13α**), 4.73 (dt, *J* = 5.0, 2.3 Hz, **H4-9α,β**), 4.62 (d, *J* = 8.3 Hz, **H1-14β**), 4.62–4.56 (m, **H4-10α,β**), 4.55 (d, *J* = 4.9 Hz, **H-X**), 4.49 (s, **H5-14β**), 4.47 (d, *J* = 6.6 Hz, **H5-9β**), 4.43–4.38 (m), 4.35 (d, *J* = 5.1 Hz, **H-X**), 4.29 (d, *J* = 2.2 Hz, **H5-15β**), 4.18 (t, *J* = 3.9 Hz, **H3-14β**), 4.14–4.08 (m, **H2-10β/H2-10α/H4**-**14β**), 4.05 (t, *J* = 4.8 Hz, **X**), 4.00 (d, *J* = 9.4 Hz, **H5**-**13α**), 3.89 (t, *J* = 9.2 Hz, **H4**-**13α**), 3.79 (dd, J = 3.4, 2.1 Hz, **H2**-**13α**), 3.76–3.63 (m, OCH_2_), 3.56–3.38 (m, OCH_2_), 1.67–1.51 (m, CH_2_), 1.46–1.22 (m, CH_2_), 0.94–0.87 (m, CH_3_).

Preparation of **H**–**C_12_ Gul** from OG

Oligoguluronate (500 mg, 2.1 mmol CO_2^−^_, 1 eq.) was dispersed in water (1.5 mL) and *n*-butanol (29 mL) in a round-bottom flask with a Dean-Stark apparatus. Methanesulfonic acid technical grade 70% wt (634 mg, 4.62 mmol, 2.2 eq.) was added and the mixture was refluxed under vigorous stirring. The water formed in the medium was gradually removed by azeotropic distillation. After 7 h, the mixture was cooled to ambient temperature. Dodecanol (1.8 mL, 8.3 mmol, 4 eq.) and the 70% methanesulfonic acid solution (288 mg, 2.1 mmol, 1 eq.) were added. The mixture was stirred at 70 °C under reduced pressure (up to 5 mbar) using distillation apparatus. Once the butanol had been completely removed (1.42 h), a 0.4N NaOH solution (18.5 mL, 7.4 mmol, 3.5 eq.) was added and the mixture was left to stir vigorously at 70 °C. for 1 h. The water was then removed by freeze-drying or by azeotropic distillation with butanol. The excess dodecanol present in the crude product was removed by solid-liquid extraction with EtOAc. At the end of this treatment, the mixture of products was dissolved in ice-cold water (27.5 mL) and EtOAc (27.5 mL), then a 1N hydrochloric acid solution (3.6 mL) was added. The aqueous solution was extracted with EtOAc (3 × 15 mL). The organic phases were combined and washed with a saturated NaC1 solution (35 mL). The organic phase was dried with MgSO_4_ and then concentrated under vacuum. A mixture of products **H**–**C_12_ Gul** was obtained (356 mg), the molar composition of which is: 15% *n*-dodecyl α-d-mannopyranosiduronic acid (**13α**), 13% *n*-dodecyl β-l-gulopyranosiduronic acid (**14β**), 26% *n*-dodecyl α-l-gulofuranosidurono-6,3-lactone (**10α**), 26% *n*-dodecyl β-l-gulofuranosidurono-6,3-lactone (**10β**), 21% *n*-dodecyl-β-l-gulofuranosiduronic acid (**15β**). ^1^H NMR (400 MHz, CD_3_OD) δ 5.07 (dd, *J* = 7.5, 4.7 Hz, **H3-10β**), 4.98 (s, **H1-10β**), 4.96 (t, *J* = 5.5 Hz, **H3-10α**), 4.92 (d, *J* = 4.5 Hz, **H1-10α**), 4.80 (d, *J* = 2.1 Hz, **H1-13α**), 4.62 (d, *J* = 8.4 Hz, **H1-14β**), 4.51 (dd, *J* = 7.6, 2.1 Hz, **H4- 15β**), 4.49 (d, *J* = 1.7 Hz, **H5-14β**), 4.39 (d, *J* = 4.3 Hz, **H5-10β**), 4.29 (d, *J* = 2.1 Hz, **H5- 15β**), 4.02 (dd, *J* = 3.6, 1.4 Hz, **H4-14β**), 4.00 (d, *J* = 9.7 Hz, **H5-13α**), 3.89 (t, *J* = 9.2 Hz, **H4-13α**), 3.84 (d, *J* = 5.0 Hz, **H2- 15β**), 3.79 (dd, *J* = 3.4, 2.1 Hz, **H2-13α**), 3.76–3.59 (m, OCH_2_), 3.56–3.36 (m, OCH_2_), 1.69–1.50 (m, CH_2_), 1.45–1.25 (m, CH_2_), 0.97–0.87 (m, CH_3_).

Preparation of **H**–**C_12_ OAlg** from OAlg

Oligoalginate (1.0 g, 2.84 mmol CO_2_^−^, 1 eq.) was dispersed in water (2.0 mL) and *n*-butanol (39 mL) in a round-bottom flask with a Dean-Stark apparatus. Methanesulfonic acid technical grade 70% wt (857 mg, 6.24 mmol, 2.2 eq.) was added and the mixture was refluxed under vigorous stirring. The water formed in the medium was gradually removed by azeotropic distillation. After 7 h, the mixture was cooled to ambient temperature. Dodecanol (2.5 mL, 11.2 mmol, 4 eq.) and the 70% methanesulfonic acid solution (391 mg, 2.85 mmol, 1 eq.) were added. The mixture was stirred at 70 °C under reduced pressure (up to 5 mbar) using distillation apparatus. Once the butanol had been completely removed (1.42 h), a 0.4N NaOH solution (25 mL) was added and the mixture was left to stir vigorously at 70 °C. for 1 h. The water was then removed by freeze-drying or by azeotropic distillation with butanol. The excess dodecanol present in the crude product was removed by solid-liquid extraction with EtOAc. At the end of this treatment, the mixture of products was dissolved in ice-cold water (30 mL) and EtOAc (45 mL), then a 1N hydrochloric acid solution (6 mL) was added. The aqueous solution was extracted with EtOAc (8 × 15 mL). The organic phases were combined and washed with a saturated NaC1 solution (60 mL) and a 1N hydrochloric acid solution (300 µL). The organic phase was dried with MgSO_4_ and then concentrated under vacuum. A mixture of products **H**–**C_12_ OAlg** was obtained (759 mg), the molar composition of which is: 23% *n*-dodecyl-α-d-mannopyranosiduronic (**13α**), 9% *n*-dodecyl-β-l-gulopyranosiduronic (**14β**), 20% *n*-dodecyl-α-l-gulofuranosidurono-6,3-lactone (**10α**), 20% *n*-dodecyl-β-l-gulofuranosidurono-6,3-lactone (**10β**), 9% *n*-dodecyl-β-l-gulofuranosiduronic acid (**15β**), 4% *n*-dodecyl-α-d-mannofuranosidurono-6.3-lactone (**9α**), 9% *n*-dodecyl-β-d-mannofuranosidurono-6.3-lactone (**9β**), 6% non-identified molecule **X**. ^1^H NMR (400 MHz, CD_3_OD) δ 5.11 (d, *J* = 6.5 Hz, **X**), 5.07 (dd, *J* = 7.5, 4.7 Hz, **H3-10β**), 4.97 (s, **H1-10β**), 4.95 (t, *J* = 5.6 Hz, **H3-10α**), 4.95 (d, *J* = 4.1 Hz, **H1-9β**), 4.91 (d, *J* = 4.5 Hz, **H1-10α**), 4.80 (d, *J* = 2.1 Hz, **H1-13α**), 4.72 (td, *J* = 4.7, 1.5 Hz, **H4-9α,β**), 4.61 (d, *J* = 8.2 Hz, **H1-14β**), 4.60–4.55 (m, **H4-10α,β**), 4.54 (d, *J* = 5.0 Hz, **X**), 4.51 (s (br), **H4-15β**), 4.48 (d, *J* = 1.6 Hz, **H5-14β**), 4.46 (d, *J* = 6.5 Hz, **H5-9β**), 4.38 (d, *J* = 4.3 Hz, **H5-10β**), 4.35 (d, *J* = 5.0 Hz, **X**), 4.29 (d, *J* = 2.0 Hz, **H5-15β**), 4.12–4.08 (m, **H2-10β/H4-10α**), 3.88 (t, *J* = 9.3 Hz, **H4-13α**), 3.78 (dd, *J* = 3.4, 2.1 Hz, **H2-13α**), 3.75–3.61 (m, OCH_2_), 3.55–3.35 (m, OCH_2_), 1.70–1.49 (m, CH_2_), 1.45–1.23 (m, CH_2_), 0.95–0.86 (m, CH_3_).

Preparation of **H**–**C_12_ s-r Alg** from s-r Alg

The semi-refined alginate derived from *Laminaria digitata* (2.0 g, 4.3 mmol sugar units, 1 eq.) was dispersed in water (60 mL) and the 70% methane-sulfonic acid solution (2.95 g, 21.5 mmol, 5 eq.) was added in a round-bottom flask with a Dean-Stark apparatus. The mixture was refluxed with vigorous stirring. At 8 h of reaction, butanol (60 mL) was added and the mixture was left at reflux with vigorous stirring. The water present in the medium was gradually removed by azeotropic distillation. After a further 15 h of reaction, and once the mixture had returned to ambient temperature, dodecanol (3.8 mL, 17 mmol, 4 eq.) was added. The mixture was stirred at 70 °C under reduced pressure (up to 5 mbar) using distillation apparatus. Once the butanol had been completely removed (1.42 h), a 0.2 N NaOH solution (60 mL) was added and the mixture was left to stir vigorously at 70 °C. for 1 h. The water was then removed by freeze-drying or by azeotropic distillation with butanol. The excess dodecanol present in the crude product was removed by solid-liquid extraction with acetone. At the end of this treatment, the mixture of products was dissolved in ice-cold water (75 mL) and then a 0.5 M oxalic acid solution (8.0 mL) was added. The water was then removed by freeze-drying. The crude product was purified by solid-liquid extraction with acetone (20 mL + 8 × 10 mL). The filtrate was concentrated under vacuum. At the end of this treatment, a mixture of products was obtained (628 mg), the weight composition of which is: 23% *n*-dodecyl-α-d-mannopyranosiduronic (**13α**), 5% *n*-dodecyl-α-d-mannofuranosidurono-6.3-lactone (**9α**), 13% *n*-dodecyl-β-d-mannofuranosidurono-6.3-lactone (**9β**), 11% *n*-dodecyl-β-l-gulopyranosiduronic (**14β**), 20% *n*-dodecyl-β-l-gulofuranosidurono-6,3-lactone (**10β**), 5% *n*-dodecyl-α-l-fucopyranosides **17α**, 8% *n*-dodecyl-β-l-fucopyranosides **17β** and 15% *n*-dodecyl-α,β-l-fucofuranosides **18α,β**. ^1^H NMR (400 MHz, acetone-d6) δ 5.08 (dd, *J* = 7.5, 4.7 Hz, **H3-10β**), 5.03 (s, **H1-10β**), 4.95 (d, *J* = 4.7 Hz, **H1-9β**), 4.89 (t, *J* = 4.8 Hz, **H3-9β**), 4.85 (d, *J* = 2.0 Hz, **H1-13α),** 4.83 (d, *J* = 3.8 Hz, **H1-17α**), 4.80 (td, *J* = 4.9, 1.4 Hz, **H4-9α,β**), 4.75 (d, *J* = 3.6 Hz, **H1-18α,β**), 4.65 (dd, *J* = 7.3, 4.4 Hz, **H4-10β**), 4.56 (d, *J* = 5.4 Hz), 4.51 (d, *J* = 1.7 Hz, **H5-14β**), 4.43 (d, *J* = 4.4 Hz, **H5-10β**), 4.27 (d, *J* = 7.7 Hz, **H1-17β**), 4.25 (t, *J* = 4.4 Hz), 4.18 (d, *J* = 4.7 Hz, **H2-10β**), 4.09 (dd, *J* = 3.6, 1.6 Hz, **H4-14β**), 3.83 (dd, *J* = 3.4, 2.0 Hz, **H2-13α**), 3.79–3.63 (m, OCH_2_), 3.51–3.31 (m, OCH_2_), 1.68–1.49 (m, CH_2_), 1.46–1.26 (m, CH_2_), 1.24 (d, *J* = 6.6 Hz, **CH_3_ 17 or 18**), 1.22 (d, *J* = 6.8 Hz, **CH_3_-17 or 18**), 1.21 (d, *J* = 6.7 Hz, **CH_3_-17** or **18**), 0.97–0.86 (m, CH_3_).

Preparation of **H**–**C_8_ crude Alg** from crude Alg

The dried milled *Ascophyllum nodosum* seaweed (100 g) was introduced into the reactor (1 L). Butanol (140 mL) and the methane-sulfonic acid solution (30 g of 70% MSA in 140 mL of butanol) were added. The mixture was refluxed and stirred with a four-blade Teflon paddle at 400 rpm. For the double envelope, the temperature was fixed at 135 °C. After 7 h of reaction, the mixture was recovered hot through the lower tap and then filtered or centrifuged to remove residues. The presence of products in the reaction medium was monitored by TLC with CH_2_Cl_2_/MeOH (95/5, *v*/*v*) as the eluent and vanillin solution as the staining reagent. The filtrate/supernatant was transferred into the reactor (1 L) and octanol (100 mL) was added. The reaction mixture is pulled under vacuum at 70 °C for 5–7 h to evaporate butanol and perform the transesterification/transglycosylation reactions. The mixture was made alkaline with a concentrated sodium hydroxide solution (5N NaOH, 20 mL). After 1–2 h at 70 °C, only non-ionic surfactants were visible by TLC (eluent: CH_2_Cl_2_/MeOH (95/5, *v*/*v*)). The anionic molecules were revealed by TLC (AcOEt/*i*PrOH/H_2_O (6/3/1, *v*/*v*/*v*)). The mixture was pulled under vacuum to evaporate the water. At room temperature, 95% of H_2_SO_4_ was added to acidify to pH 2–3 and precipitate the salts (counter-ions of the anionic surfactants). Surfactants were miscible in octanol. This oil phase was filtered to remove the salts which were insoluble. The oily filtrate was injected into a molecular distillation apparatus to eliminate octanol. At 100 °C under reduced pressure (1 mbar), 24 g of the surfactant composition was recovered in the form of a viscous syrup, and consequently with a mass yield of approximately 24% relative to the starting algae. At room temperature, the product is a pasty and hygroscopic solid. The mixture was submitted to thermogravimetric analysis, which showed that it consisted of 83.2% organic matter; 10.5% water; and 6.75% ash. ^1^H NMR analysis revealed the presence of 30 mol% *n*-octyl α-d-mannopyranosiduronic acid **19α**, 6 mol% *n*-octyl β-l-gulopyranosiduronic acid **20β**, *n*-octyl α-l-gulofuranosidurono-6,3-lactone **21α**, 13 mol% *n*-octyl-α-l-fucopyranoside **22α**, 9 mol% *n*-octyl-β-l-fucopyranosides **22β**, 9 mol% *n*-ocyl-α-l-fucofuranosides **23α** and 28 mol% *n*-octyl-β-l-fucofuranoside **23β**. ^1^H NMR (400 MHz, CD_3_OD) δ 5.05 (d, *J* = 3.2 Hz), 4.97 (t, *J* = 5.5 Hz, **H5-21α**), 4.93 (d, *J* = 4.6 Hz, **H1-21α**), 4.82 (d, *J* = 2.0 Hz, **H1-19α**), 4.78 (d, *J* = 3.8 Hz, **H1-22α**), 4.75 (d, *J* = 2.2 Hz, **H1-23β**), 4.72 (d, *J* = 3.7 Hz, **H1-23α**), 4.64 (d, *J* = 8.1 Hz, **H1-20β**), 4.61 (d, *J* = 4.6 Hz), 4.50 (d, *J* = 1.6 Hz, **H5-20β**), 4.43 (t, *J* = 4.8 Hz), 4.26 (d, *J* = 7.8 Hz, **H1-22β**), 3.88 (t, *J* = 9.4 Hz, **H4-19α**), 3.80 (dd, *J* = 3.5, 1.9 Hz, **H2-19α**), 3.77–3.63 (m, OCH_2_), 3.53–3.35 (m, OCH_2_), 1.72–1.50 (m, CH_2_), 1.48–1.30 (m, CH_2_), 1.27 (d, *J* = 6.4 Hz, **CH_3_ 22 or 18**), 1.26 (d, *J* = 6.5 Hz, **CH_3_-22 or 23**), 1.22 (d, *J* = 6.6 Hz, **CH_3_-22 or 23**), 1.19 (d, *J* = 6.5 Hz, **CH_3_-22 or 23**), 1.01–0.88 (m, CH_3_).

### 4.2. Physico-Chemistry

Critical micelle concentrations (CMC) and interfacial tensions (IFT) were measured on a force tensiometer Krüss K100. The critical micelle concentrations (CMC) were determined using the du Noüy ring method and the Krüss Laboratory Desktop software with the Surfactant Characteristics program. A water solution of surfactant was prepared around 10 g/L. Only 10 mL of this solution was used for the CMC determination. The deionized water was added thanks to an automatic burette controlled by the software. The concentration and tension determination were automatically determined by the software. The CMC was measured at 25 °C.

## 5. Conclusions

In this study, it has been proven for the first time that the conversion of oligo- and polysaccharide alginates in refined or semi-refined forms into biodegradable alkyl uronate surfactants can be carried out by one-pot acid hydrolysis, butanolysis, transesterification, transacetalisation, and saponification reactions, thus avoiding the isolation of any reaction intermediates. In addition, an in situ process was developed to manufacture surfactant compositions directly from crude milled brown seaweeds without requiring the standard steps of polysaccharide extraction and purification. These alkyl uronate monosaccharides as isomeric mixtures, with or without alkyl glycoside co-products, exhibit attractive surface activity and reduced aquatic ecotoxicity compared to commercial sodium laureth sulphate. Further physicochemical studies to evaluate the potential of these innovative surfactant compositions in cosmetic formulations are currently under investigation. Moreover, the transposition of the strategy, which consists in using crude vegetable raw materials instead of extracted polysaccharides, was also successfully achieved in the case of sugar beet pulps containing pectins [20,26]: this work, which is not described in this article, will be submitted for publication elsewhere.

## Figures and Tables

**Table 1:** Characteristics of oligomannuronate (OM), oligoguluronate (OG) [16,17], oligoalginate (OAlg), and semi-refined alginates (s-r Alg) [18].

	Description	Method	Result	Units
Oligomannuronate (OM)	Solids	Constant weight at 103 °C	95.6	%dry/crude
	Mineral matter	12 h, 550 °C	31.9	%dry/crude
	Ratio (M/G)	By calculation, ^1^H NMR	2.7	
	DP	By calculation	4.0	
Oligoguluronate (OG)	Solids	Constant weight at 103 °C	100.0	%dry/crude
	Mineral matter	12 h, 550 °C	26.3	%dry/crude
	Ratio (M/G)	By calculation, ^1^H NMR	0.04	
	DP	By calculation	30.0	
Oligoalginate (OAlg)	Solids	Constant weight at 103 °C	90.1	%dry/crude
	Mineral matter	12 h, 550 °C	44.3	%dry/crude
	Ratio (M/G)	By calculation, ^1^H NMR	1.4	
	DP	By calculation	12.7	
Semi-refined alginates (s-r Alg)	Solids	Constant weight at 103 °C	94.9	%dry/crude
	Mineral matter	12 h, 550 °C	47.0	%dry/crude
	Mannuronic and guluronic content	Methanolysis	29.2	%dry/crude
	Glucose content	Methanolysis	10.9	%dry/crude
	Xylose content	Methanolysis	<0.5	%dry/crude
	Fucose content	Methanolysis	2.1	%dry/crude
	Ratio (M/G)	By calculation, ^1^H NMR	2.6	

**Table 2:** ^1^H NMR (CDCl_3_, 400 MHz) characteristics of the butylated lactone, furanose, and pyranose mannuronate **1**–**3** and guluronate **4**–**5** monomers identified on the crude mixture C_4_–C_4_ Man and/or in the fractions isolated after column chromatography.

Compounds	δ H_1_ (ppm) (Multiplicity)	*J*_1,2_ (Hz)	Form	Composition (mol%)
**1α** (Man)	4.91 (d)	1.7	α-Pyranose	52
**2α** (Man)	4.97 (s)	-	α-Furanose	7
**3α** (Man)	5.05 (d)	2.0	α-Lactone	11
**3β** (Man)	5.03 (d)	4.6	β-Lactone	13
**4α** (Gul)	4.95 (d)	4.4	α-Lactone	5
**4β** (Gul)	5.06 (s)	-	β-Lactone	5
**5β** (Gul)	4.91 (s)	-	β-Furanose	3

**Table 3:** Yield and/or mass of butyl mannuronate **1**–**3** and guluronate **4**–**5** monomers, as well as furan derivative **6** isolated after column chromatography, depending on MSA quantity. Reaction conditions: crude OM = 500 mg (OM = 326 mg), n_uronate unit_ = 1.81 mmol, BuOH = 25 mL_,_ H_2_O (1.7–2 mL/g of crude OM) 140 °C, 7 h.

	MSA (*n* eq)			
**Compounds**	1.7	2.2	2.6	4.7
**1α**	183 mg (yld = 33%)	132 mg (yld = 24%)	150 mg (yld = 27%)	69 mg (yld = 12%)
**2α, 3α,β, 4α,β, 5β**	126 mg	118 mg	117 mg	31 mg
**6**	57 mg	166 mg	148 mg	380 mg

**Table 4:** Yield and/or mass of dodecyl mannuronate and guluronate monomers **7**–**12** isolated after two successive column chromatographies, depending on DodOH and MSA quantities. Reaction conditions: (i) crude OM = 500 mg (OM = 326 mg), n_uronate unit_ = 1.81 mmol, MSA = 1.7 eq, BuOH = 25 mL_,_ H_2_O (1.7–2 mL/g of crude OM), 140 °C, 7 h; (ii) DodOH (*n’* eq), MSA (*n* eq), 5 mbar, 2.17 h. ^a^ Reaction time for the second step = 1.75 h.

Entries	DodOH (*n’* eq)	MSA (*n* eq)	7α	8α, 9α,β, 10α,β, 11α,β, 12β
**1 ^a^**	16	2	229 mg (yld = 24%)	153 mg
**2**	4	1	240 mg (yld = 25%)	168 mg
**3**	4	0.5	200 mg (yld = 21%)	184 mg

**Table 5:** ^1^H NMR (CDCl_3_, 400 MHz) characteristics of the dodecyl lactone, furanose, and pyranose mannuronate **7**–**9** and guluronate **10**–**12** monomers identified in the crude mixture **C_12_**–**C_12_ Man** (after first column chromatography). n.d. = not detectable/determined.

Compounds	δ H_1_ (ppm) (Multiplicity)	*J*_1,2_ (Hz)	Form	Composition (mol%)
**7α** (Man)	4.92 (d)	1.7	α-Pyranose	48
**8α** (Man)	4.97 (s)	-	α-Furanose	9
**9α** (Man)	5.05 (d)	2.0	α-Lactone	5
**9β** (Man)	5.03 (d)	4.5	β-Lactone	22
**10α** (Gul)	4.95 (d)	4.5	α-Lactone	3
**10β** (Gul)	5.07 (s)	-	β-Lactone	3
**11β** (Gul) *	n.d.	n.d.	β-Pyranose	5
**12β** (Gul)	4.91 (s)	-	β-Furanose	6

* Characteristic signal: δ H_5_ = 4.52 ppm (d), *J* = 1.0 Hz.

**Table 6:** ^1^H NMR (CD_3_OD, 400 MHz) characteristics of the C_12_ lactone, furanose, and pyranose mannuronate **9**,**13** and guluronate **10**,**14**,**15** monomers present in the crude mixture **H**–**C_12_ Man**. n.d. = not detectable/determined.

Compounds	δ H_1_ (ppm) (Multiplicity)	*J*_1,2_ (Hz)	Form	Composition (mol%)
**13α** (Man)	4.80 (d)	2.1	α-Pyranose	47
**9α** (Man)	5.00 (d)	1.6	α-Lactone	4
**9β** (Man)	4.96 (d)	3.8-	β-Lactone	12
**14β** (Gul)	4.62 (d)	8.3	β-Pyranose	4
**10α** (Gul)	4.92 (d)	4.4	α-Lactone	9
**10β** (Gul)	4.98 (s)	-	β-Lactone	9
**15β** (Gul) *	n.d.	n.d.	β-Furanose	4

* Characteristic signal: δ H_5_ = 4.29 ppm (d), *J* = 2.2 Hz.

**Table 7:** ^1^H NMR (CD_3_OD, 400 MHz) characteristics of the C_12_ lactone, furanose, and pyranose mannuronate **13** and guluronate **10**,**14**,**15** monomers identified in the crude mixture **H**–**C_12_ Gul**. n.d. = not detectable/determined.

Compounds	δ H_1_ (ppm) (Multiplicity)	*J*_1,2_ (Hz)	Form	Composition (mol%)
**13α** (Man)	4.80 (d)	2.1	α-Pyranose	15
**14β** (Gul)	4.62 (d)	8.4	β-Pyranose	13
**10α** (Gul)	4.92 (d)	4.5	α-Lactone	26
**10β** (Gul)	4.98 (s)	-	β-Lactone	26
**15β** (Gul) *	n.d.	n.d.	β-Furanose	21

* Characteristic signal: δ H_5_ = 4.29 ppm (d), *J* = 2.0 Hz.

**Table 8:** ^1^H NMR (CD_3_OD, 400 MHz) characteristics of the C_12_ lactone, furanose, and pyranose mannuronate **9**,**13** and guluronate **10**,**14**,**15** monomers identified in the crude mixture **H**–**C_12_ OAlg**. n.d. = not detectable/determined.

Compounds	δ H_1_ (ppm) (Multiplicity)	*J*_1,2_ (Hz)	Form	Composition (mol%)
**13α** (Man)	4.80 (d)	2.1	α-Pyranose	23
**9α** (Man) *	n.d.	n.d.	α-Lactone	4
**9β** (Man)	4.95 (d)	4.1-	β-Lactone	9
**14β** (Gul)	4.61 (d)	8.2	β-Pyranose	9
**10α** (Gul)	4.91 (d)	4.5	α-Lactone	20
**10β** (Gul)	4.97 (s)	-	β-Lactone	20
**15β** (Gul) **	n.d.	n.d.	β-Furanose	9

* Characteristic signal: δ H_4_ = 4.72 ppm (td), *J* = 4.7, 1.5 Hz; ** Characteristic signal: δ H_5_ = 4.29 ppm (d), *J* = 2.0 Hz.

**Table 9:** ^1^H NMR (acetone-d6, 400 MHz) characteristics of the C_12_ lactone, furanose, and pyranose mannuronate **9**,**13** and guluronate **10,14** monomers in addition to C_12_ fucosides **17**,**18** identified in the crude mixture **H**–**C_12_ s-r Alg**. n.d. = not detectable/determined.

Compounds	δ H_1_ (ppm) (Multiplicity)	*J*_1,2_ (Hz)	Form	Composition (mol%)
**13α** (Man)	4.85 (d)	2.0	α-Pyranose	23
**9α** (Man) *	n.d.	n.d.	α-Lactone	5
**9β** (Man)	4.95 (d)	4.7-	β-Lactone	13
**14β** (Gul) **	n.d.	n.d.	β-Pyranose	11
**10β** (Gul)	5.03 (s)	-	β-Lactone	20
**17α** (Fuc)	4.83 (d)	3.8	α-Pyranose	5
**17β** (Fuc)	4.27 (d)	7.7	β-Pyranose	8
**18α** (Fuc)	4.75 (d)	3.6	α-Furanose	(+18β) 15
**18β** (Fuc)	4.75 (d)	n.d.	β-Furanose	(+18α) 15

* Characteristic signal: δ H_4_ = 4.80 ppm (td), *J* = 4.9, 1.5 Hz; ** Characteristic signal: δ H_5_ = 4.51 ppm (d), *J* = 1.7 Hz.

**Table 10:** ^1^H NMR (CD_3_OD, 400 MHz) characteristics of the C_8_ lactone, furanose, and pyranose mannuronate **19** and guluronate **20**,**21** monomers in addition to C_8_ fucosides **22**,**23** identified in the mixture **H**–**C_8_ crude Alg** derived from brown seaweeds.

Compounds	δ H_1_ (ppm) (Multiplicity)	*J*_1,2_ (Hz)	Form	Composition (mol%)
**19α** (Man)	4.82 (d)	2.0	α-Pyranose	30
**20β** (Gul)	4.64 (d)	8.1	β-Pyranose	6
**21α** (Gul)	4.93 (d)	4.6	α-Lactone	5
**22α** (Fuc)	4.78 (d)	3.8	α-Pyranose	13
**22β** (Fuc)	4.26 (d)	7.8	β-Pyranose	9
**23α** (Fuc)	4.72 (d)	3.7	α-Furanose	9
**23β** (Fuc)	4.75 (d)	2.2	β-Furanose	28

**Table 11:** Classification of substances that may have acute toxicity in the aquatic environment.

**Category Acute 1 (very toxic)**	
**CL_50_ 96 h** (for the fish)	≤1 mg L^−1^ and/or
**CE_50_ 48 h** (for the shellfish)	≤1 mg L^−1^ and/or
**CEr_50_ 72 h** (for the seaweed)	≤1 mg L^−1^
**Category Acute 2 (toxic)**	
**CL_50_ 96 h** (for the fish)	>1 but ≤10 mg L^−1^ and/or
**CE_50_ 48 h** (for the shellfish)	>1 but ≤10 mg L^−1^ and/or
**CEr_50_ 72 h** (for the seaweed)	>1 but ≤10 mg L^−1^
**Category Acute 3 (poorly toxic)**	
**CL_50_ 96 h** (for the fish)	>10 but ≤100 mg L^−1^ and/or
**CE_50_ 48 h** (for the shellfish)	>10 but ≤100 mg L^−1^ and/or
**CEr_50_ 72 h** (for the seaweed)	>10 but ≤100 mg L^−1^
**Above 100 mg L^−1^, the substance is considered as non-toxic**	

**Table 12:** Results of ecotoxicity tests on **SLES** and **H**–**C_12_ s-r Alg**.

Test	Effect	Toxicological Descriptor *	SLES	H–C_12_ s-r Alg
Microalgae	Rate of growth	CEr_50_—72 h	7.0 mg L^−1^ **Toxic**	45.8 mg L^−1^ **Poorly toxic**
Daphnia	Immobilization	CE_50_—24 h	11.6 mg L^−1^ **Poorly toxic**	>100 mg L^−1^ **Non-toxic**
Fish	Mortality	CL_50_—96 h	11.2 mg L^−1^ **Poorly toxic**	67.0 mg L^−1^ **Poorly toxic**

* The toxicological descriptors C X—T correspond to the concentrations causing an effect on X % of the population after a time T. NOEC (No observed effect concentration) is the highest concentration in the range of tests performed that do not cause significant effects on organisms.

## Data Availability

The data that support the findings of this study are available from the corresponding author upon reasonable request.

## Supplementary Materials

The following are available online: https://www.mdpi.com/article/10.3390/molecules28135201/s1, Figure S1: ^1^H NMR spectrum of **C_4_**–**C_4_ Man** from OM (δ: 7.2–0.6 ppm) (400 MHz, CDCl_3_); Figure S2: Zooms of ^1^H NMR spectrum of **C_4_**–**C_4_ Man** from OM in the zones: (a) 7.2–5.5 ppm; (b) 5.16–4.68 ppm; (c) 4.50–3.60 ppm (400 MHz, CDCl_3_); Figure S3: Zooms of ^1^H NMR spectra of **fractions F1, F2 and F3** isolated after column chromatography of **C_4_**–**C_4_ Man** from OM (400 MHz, CDCl_3_); Figure S4: Zooms of ^1^H NMR spectra of **fractions F4 and F5** isolated after column chromatography of **C_4_**–**C_4_ Man** from OM (400 MHz, CDCl_3_); Figure S5: ^1^H NMR spectrum of **C_12_**–**C_12_ Man** from OM (δ: 0.6–2.2 ppm) (400 MHz, CDCl_3_); Figure S6: Zooms of ^1^H NMR spectrum of **C_12_**–**C_12_ Man** from OM in the zones: (a) 5.15–4.50 ppm; (b) 4.50–3.86 ppm (400 MHz, CDCl_3_); Figure S7: Zooms of ^1^H NMR spectra of **fractions F1, F2 and F3** isolated after the second column chromatography of **C_12_-**–**C_12_ Man** from OM (400 MHz, CDCl_3_); Figure S8: Zoom of ^1^H NMR spectrum of **fraction F4** isolated after column chromatography of **C_12_**–**C_12_ Man** from OM (400 MHz, CDCl_3_); Figure S9: ^1^H NMR spectrum of **H**–**C_12_ Man** from OM (δ: 5.2–0.6 ppm) (400 MHz, CD_3_OD); Figure S10: Zooms of ^1^H NMR spectrum of **H**–**C_12_ Man** from OM in the zones: (a) 5.15–4.35 ppm; (b) 4.35–3.77 ppm (400 MHz, CD_3_OD); Figure S11: ^1^H NMR spectrum of **H**–**C_12_ Gul** from OG (δ: 5.2–0.6 ppm) (400 MHz, CD_3_OD); Figure S12: Zooms of ^1^H NMR spectrum of **H**–**C_12_ Gul** from OG in the zones: (a) 5.15–4.35 ppm; (b) 4.32–3.76 ppm (400 MHz, CD_3_OD); Figure S13: ^1^H NMR spectrum of **H**–**C_12_ OAlg** from OAlg (δ: 5.2–0.6 ppm) (400 MHz, CD_3_OD); Figure S14: Zooms of ^1^H NMR spectrum of **H**–**C_12_ OAlg** from OAlg in the zones: (a) 5.15–4.35 ppm; (b) 4.32–3.74 ppm (400 MHz, CD_3_OD); Figure S15: ^1^H NMR spectrum of **H**–**C_12_ s-r Alg** from s-r Alg (δ: 5.2–0.6 ppm) (400 MHz, acetone-d6); Figure S16: Zooms of ^1^H NMR spectrum of **H**–**C_12_ s-r Alg** from s-r Alg in the zones: (a) 5.10–4.40 ppm; (b) 4.34–3.81 ppm; (c) 1.75–0.80 ppm (400 MHz, acetone-d6); Figure S17: ^1^H NMR spectrum of **H**–**C_8_ crude Alg** from crude Alg (δ: 5.20–3.80 ppm) (400 MHz, CD_3_OD); Figure S18: Zoom of ^1^H NMR spectrum of **H**–**C_8_ crude Alg** from crude Alg in the zone 1.76–0.85 (400 MHz, CD_3_OD); Figure S19: Biodegradability results (**H**–**C_12_ s-r Alg**) according to the OCDE 301 B method; Figure S20: Biodegradability results (**SLES**) according to the OCDE 301 B method.

## Sample Availability

Samples of the compounds are not available from the authors.
