# Supplementary material for: Cascading One-Pot Synthesis of Biodegradable Uronic Acid-Based Surfactants from Oligoalginates, Semi-Refined Alginates, and Crude Brown Seaweeds
Source: Molecules. 2023 Jul 4;28(13):5201. doi: 10.3390/molecules28135201 (PMC10343331; doi:10.3390/molecules28135201)
Supplement: Supplementary file 1 [file molecules-28-05201-s001.zip › molecules-2448928-supplementary.pdf]

Supplementary Materials :

Cascading One-opt Synthesis of Biodegradable Uronic Acid-based Surfactants from Oligoalginates, Semi-Refined Alginates and Crude Brown Seaweeds

Freddy Pessel, Guillaume Noirbent, Cédric Boyère, Sacha Pérocheau Arnaud, Tiphaine Wong, Laura Durand, and Thierry Benvegnu*

Contents:

Figure S1 ^{1}H NMR spectrum of **C_4-C_4 Man** from OM (δ: 7.2-0.6 ppm) (400 MHz, $CDCl_3$) . **3**

Figure S2 Zooms of ^{1}H NMR spectrum of **C_4-C_4 Man** from OM in the zones: (a) 7.2-5.5 ppm; (b) 5.16-4.68 ppm; (c) 4.50-3.60 ppm (400 MHz, $CDCl_3$) . **4**

Figure S3 Zooms of ^{1}H NMR spectra of **fractions F1, F2 and F3** isolated after column chromatography of **C_4-C_4 Man** from OM (400 MHz, $CDCl_3$) . **5**

Figure S4 Zooms of ^{1}H NMR spectra of **fractions F4 and F5** isolated after column chromatography of **C_4-C_4 Man** from OM (400 MHz, $CDCl_3$) **6**

Figure S5 ^{1}H NMR spectrum of **C_{12}-C_{12} Man** from OM (δ: 0.6 -2.2 ppm) (400 MHz, $CDCl_3$) **7**

Figure S6 Zooms of ^{1}H NMR spectrum of **C_{12}-C_{12} Man** from OM in the zones: (a) 5.15-4.50 ppm; (b) 4.50-3.86 ppm (400 MHz, $CDCl_3$) **8**

Figure S7 Zooms of ^{1}H NMR spectra of **fractions F1, F2 and F3** isolated after the second column chromatography of **C_{12}-C_{12} Man** from OM (400 MHz, $CDCl_3$) **9**

Figure S8 Zoom of ^{1}H NMR spectrum of **fraction F4** isolated after column chromatography of **C_{12}-C_{12} Man** from OM (400 MHz, $CDCl_3$) **10**

Figure S9 ^{1}H NMR spectrum of **H-C_{12} Man** from OM (δ: 5.2-0.6 ppm) (400 MHz, CD_3OD) **11**

Figure S10 Zooms of ^{1}H NMR spectrum of **H-C_{12} Man** from OM in the zones: (a) 5.15-4.35 ppm; (b) 4.35-3.77ppm (400 MHz, CD_3OD) **12**

Figure S11 ^{1}H NMR spectrum of **H-C_{12} Gul** from OG (δ: 5.2-0.6 ppm) (400 MHz, CD_3OD) **13**

Figure S12 Zooms of ^{1}H NMR spectrum of **H-C_{12} Gul** from OG in the zones: (a) 5.15-4.35 ppm; (b) 4.32-3.76 ppm (400 MHz, CD_3OD) **14**

Figure S13 ^{1}H NMR spectrum of **H-C_{12} OAlg** from OAlg (δ: 5.2-0.6 ppm) (400 MHz, CD_3OD) **15**

Figure S14: Zooms of ^{1}H NMR spectrum of **H-C_{12} OAlg** from OAlg in the zones: (a) 5.15-4.35 ppm; (b) 4.32-3.74 ppm (400 MHz, CD_3OD) **16**

Figure S15 ^{1}H NMR spectrum of **H-C_{12} s-r Alg** from s-r Alg (δ: 5.2-0.6 ppm) (400 MHz, acetone-d6) **17**

Figure S16 Zooms of ^{1}H NMR spectrum of **H-C_{12} s-r Alg** from s-r Alg in the zones: (a) 5.10-4.40 ppm; (b) 4.34-3.81 ppm; (c) 1.75-0.80 ppm (400 MHz, acetone-d6) **18**

Figure S17 ^{1}H NMR spectrum of **H-C_8 crude Alg** from crude Alg (δ: 5.20-3.80 ppm) (400 MHz, CD_3OD) **19**

Figure S18 Zoom of ^{1}H NMR spectrum of **H-C_8 crude Alg** from crude Alg in the zone 1.76-0.85 (400 MHz, CD_3OD) **19**

Figure S19 Biodegradability results (**H-C12 s-r Alg**) according to the OCDE 301 B method **20**

Figure S20 Biodegradability results (**SLES**) according to the OCDE 301 B method

20

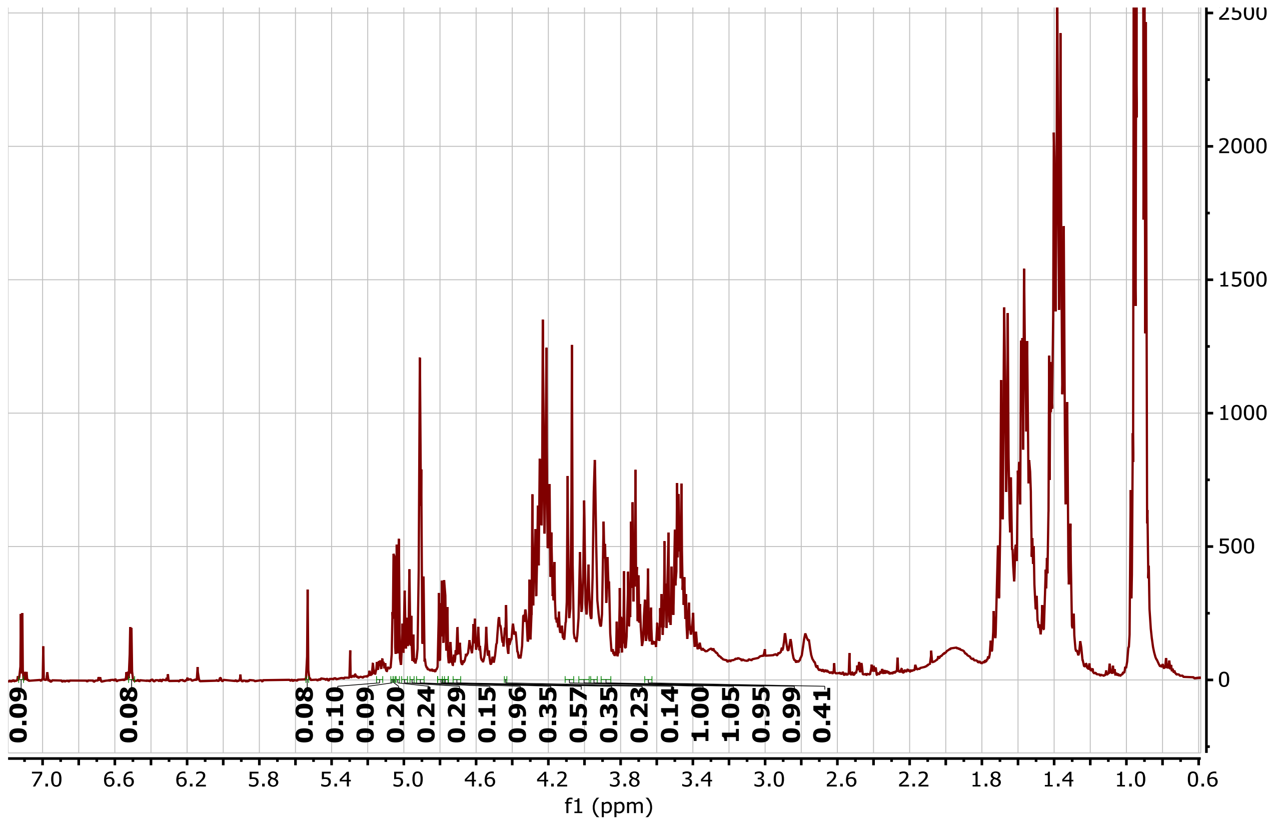

Figure S1: ^{1}H NMR spectrum of **C_4-C_4 Man** from OM (δ: 7.2-0.6 ppm) (400 MHz, $CDCl_3$).

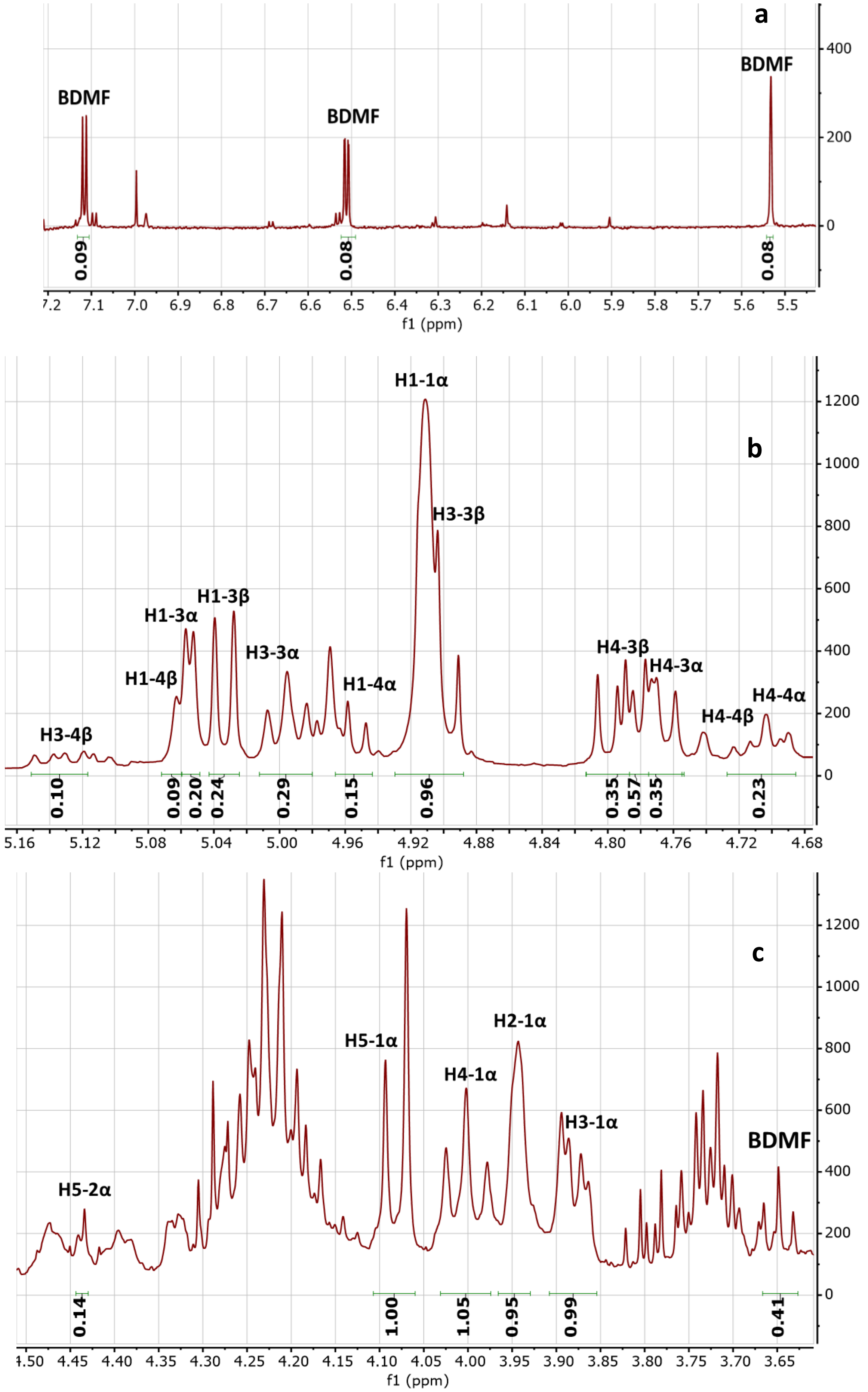

Figure S2: Zooms of ^{1}H NMR spectrum of **C_4-C_4 Man** from OM in the zones: (a) 7.2-5.5 ppm; (b) 5.16-4.68 ppm; (c) 4.50-3.60 ppm (400 MHz, $CDCl_3$).

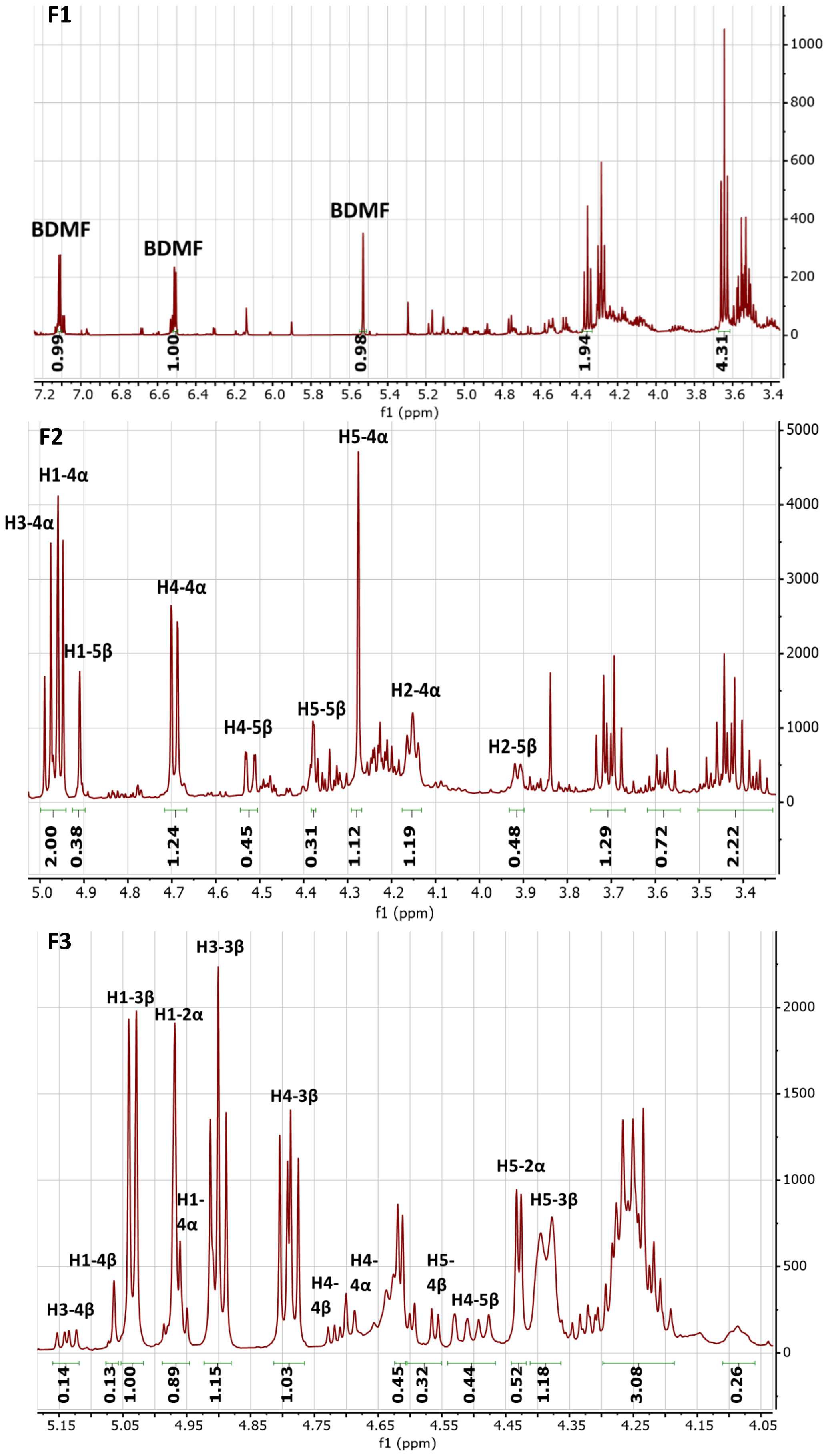

Figure S3: Zooms of ^{1}H NMR spectra of **fractions F1, F2 and F3** isolated after column chromatography of **C_4-C_4 Man** from OM (400 MHz, $CDCl_3$).

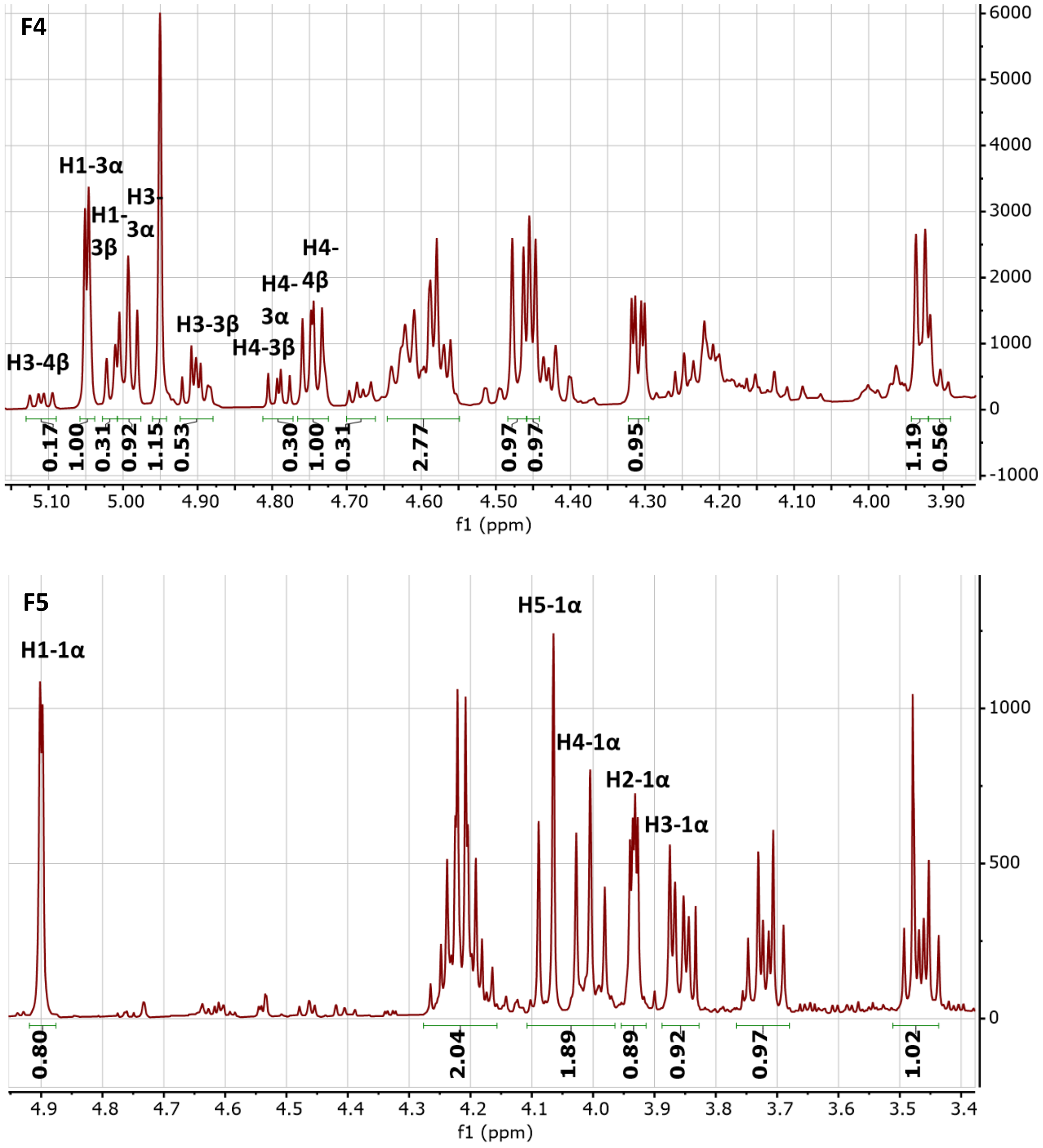

Figure S4: Zooms of ^{1}H NMR spectra of **fractions F4 and F5** isolated after column chromatography of **C_4-C_4 Man** from OM (400 MHz, $CDCl_3$).

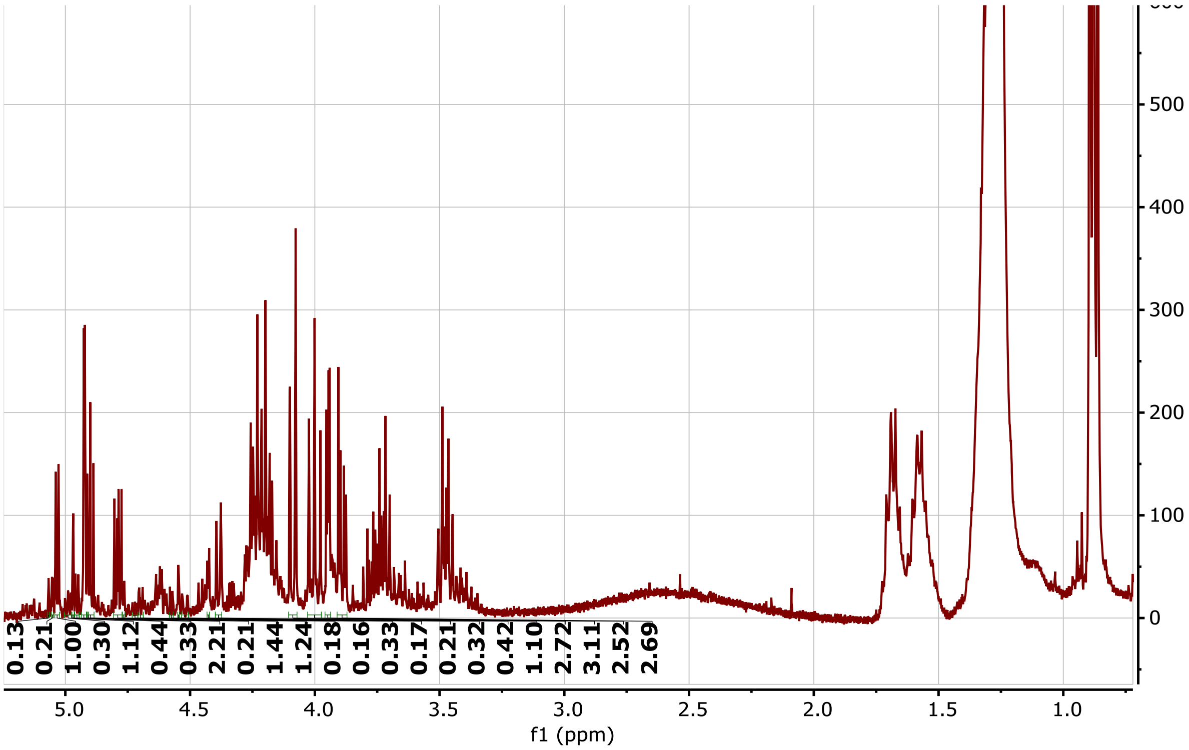

Figure S5: ^{1}H NMR spectrum of **C$_{12}$-C$_{12}$ Man** from OM (δ: 5.2-0.6 ppm) (400 MHz, $CDCl_3$).

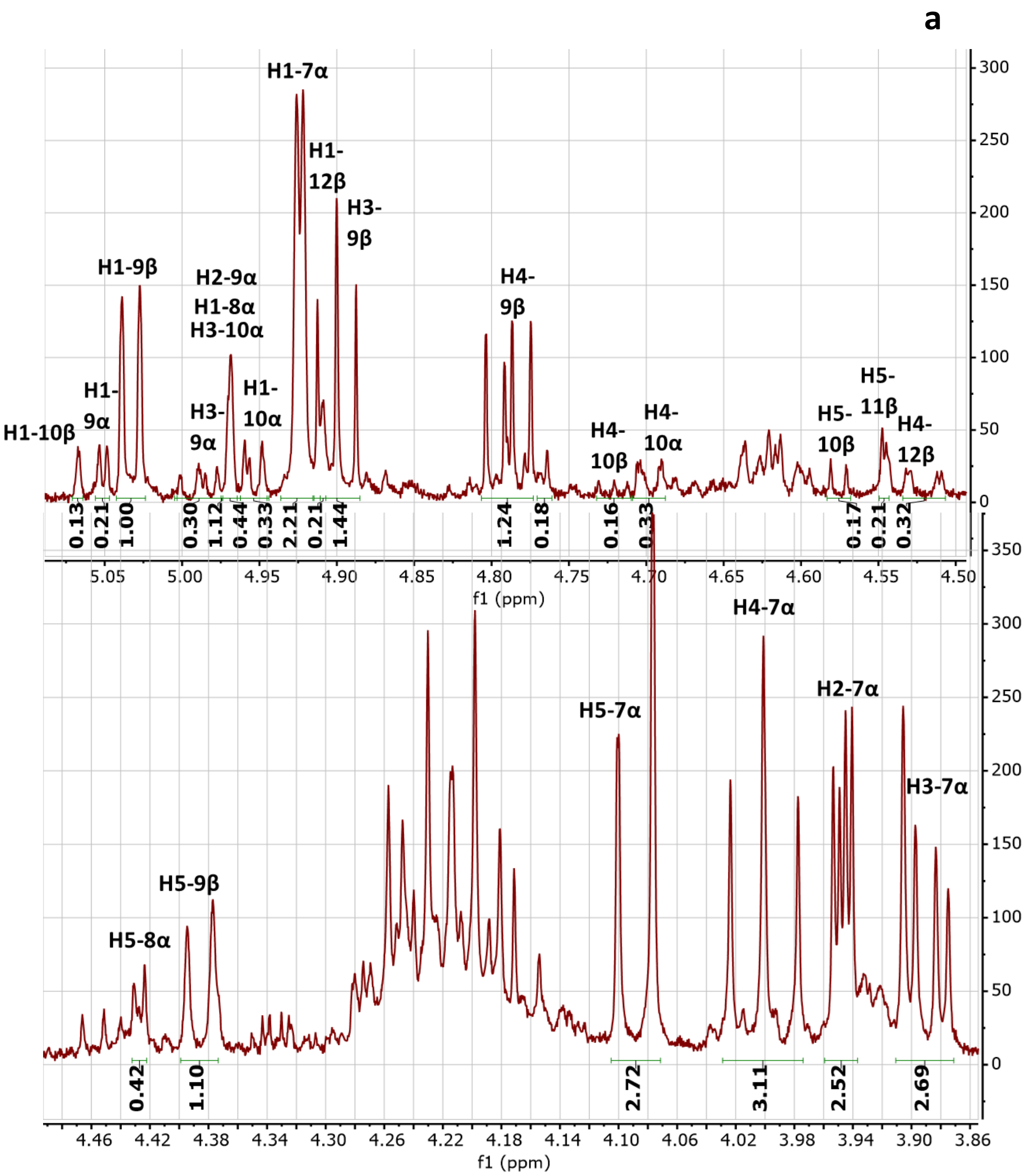

Figure S6: Zooms of ^{1}H NMR spectrum of **C12-C12 Man** from OM in the zones: (a) 5.15-4.50 ppm; (b) 4.50-3.86 ppm (400 MHz, $CDCl_3$).

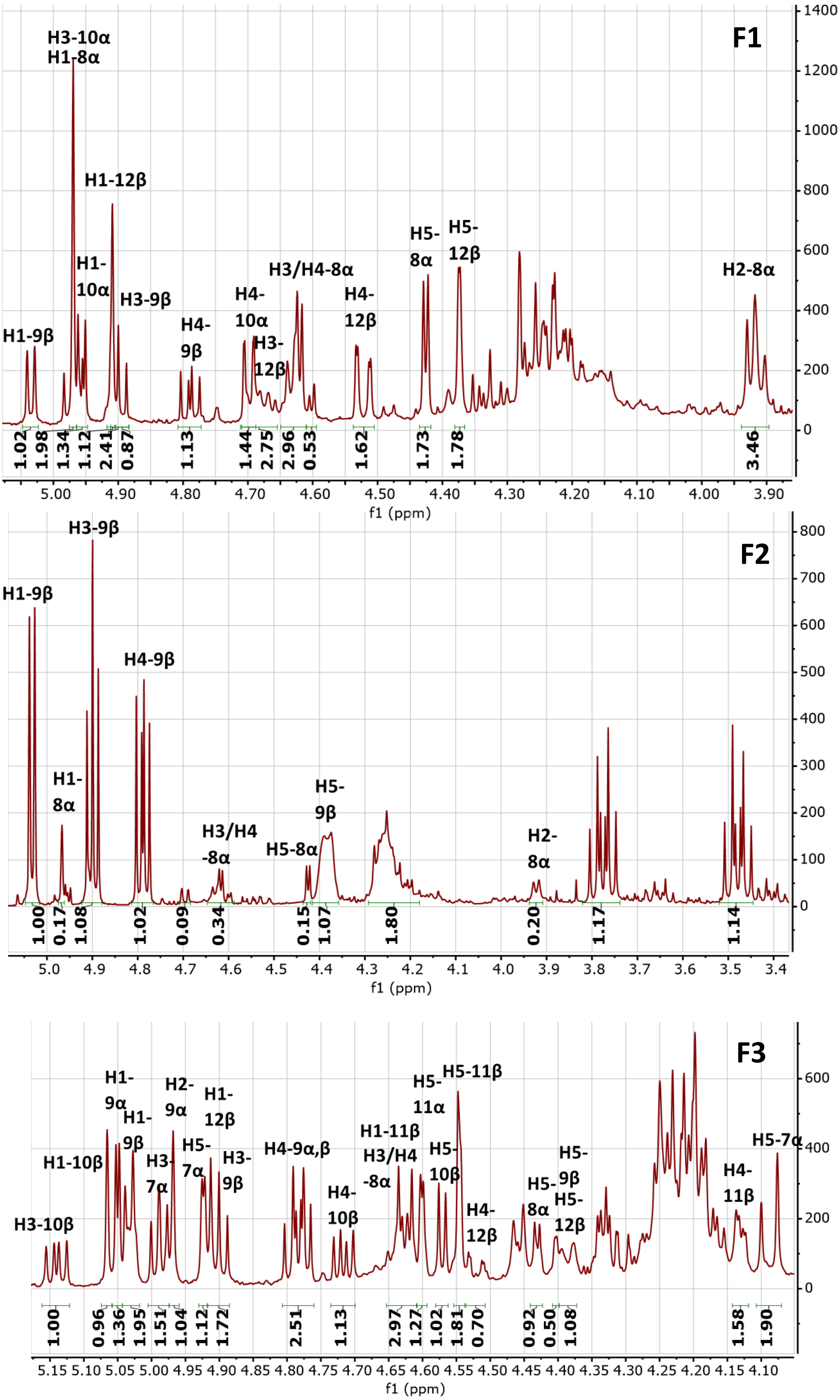

Figure S7: Zooms of ^{1}H NMR spectra of **fractions F1, F2 and F3** isolated after the second column chromatography of **C_{12}-C_{12} Man** from OM (400 MHz, $CDCl_3$).

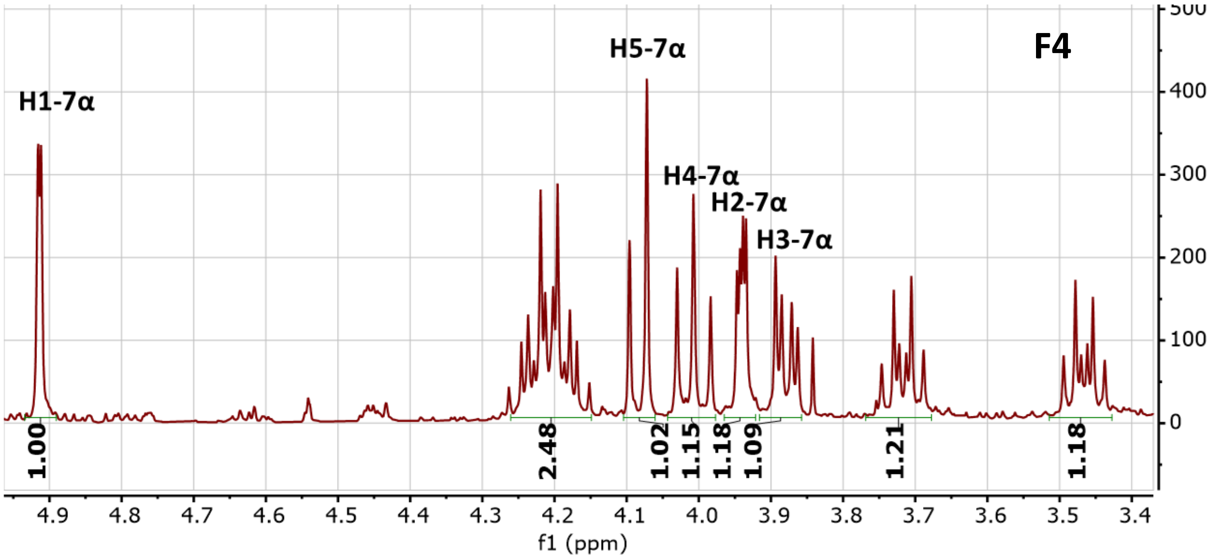

Figure S8: Zoom of ^{1}H NMR spectrum of **fraction F4** isolated after column chromatography of **C_{12}-C_{12} Man** from OM (400 MHz, $CDCl_3$).

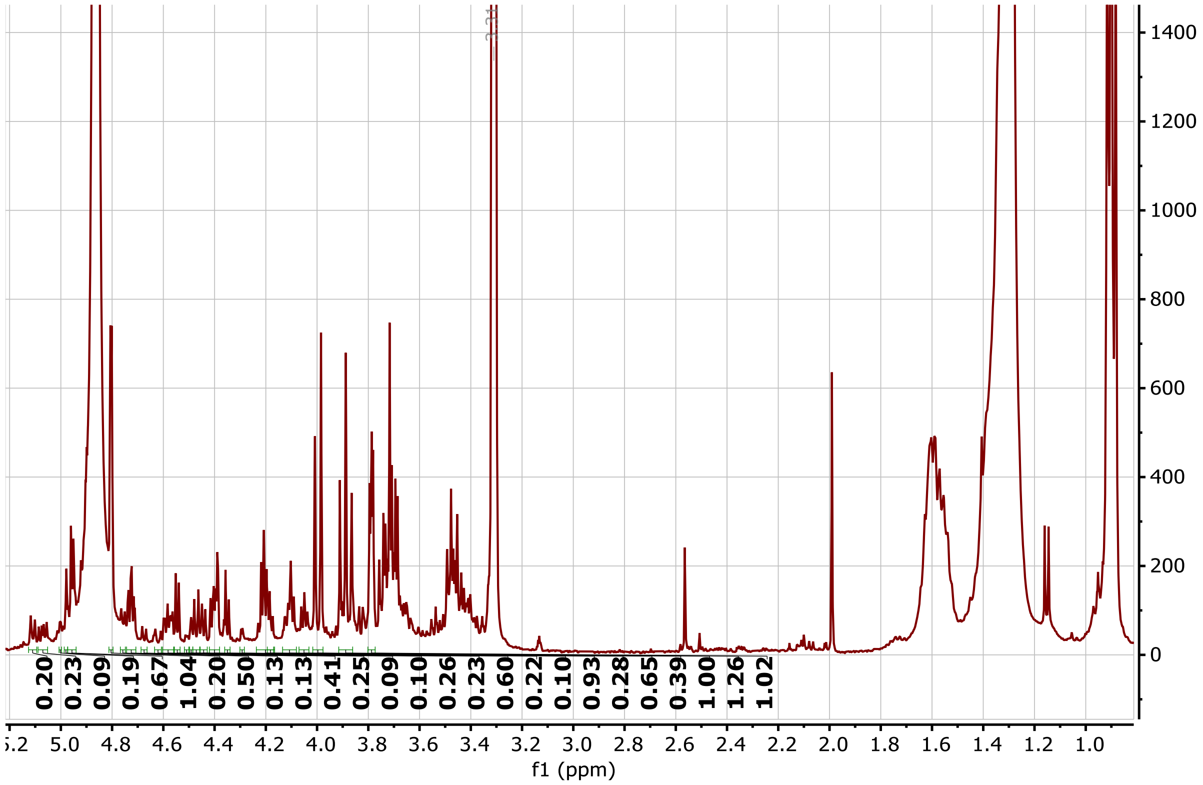

Figure S9: ^{1}H NMR spectrum of **H-C$_{12}$ Man** from OM (δ: 5.2-0.6 ppm) (400 MHz, CD_3OD).

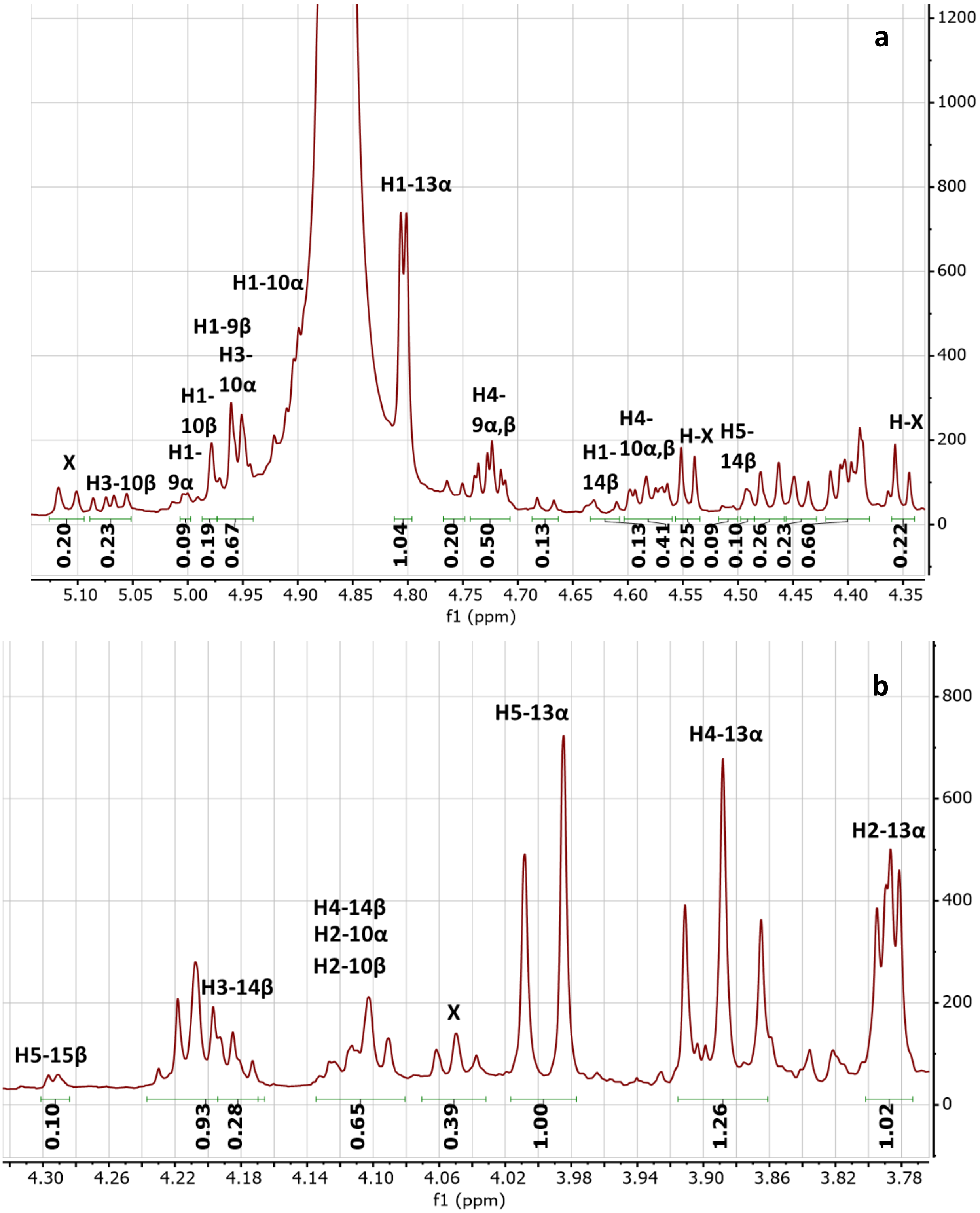

Figure S10: Zooms of ^{1}H NMR spectrum of **H-C$_{12}$ Man** from OM in the zones: (a) 5.15-4.35 ppm; (b) 4.35-3.77ppm (400 MHz, CD_3OD).

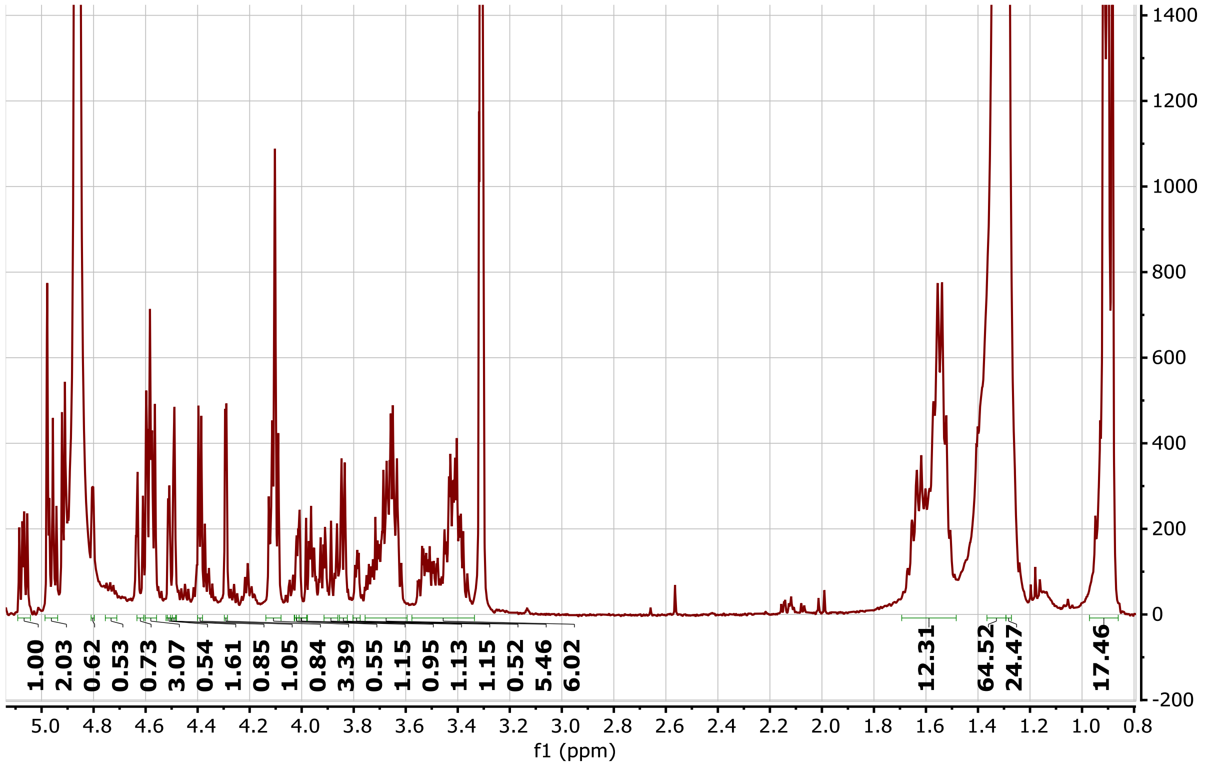

Figure S11: ^{1}H NMR spectrum of **H-C$_{12}$ Gul** from OG (δ: 5.2-0.6 ppm) (400 MHz, CD_3OD).

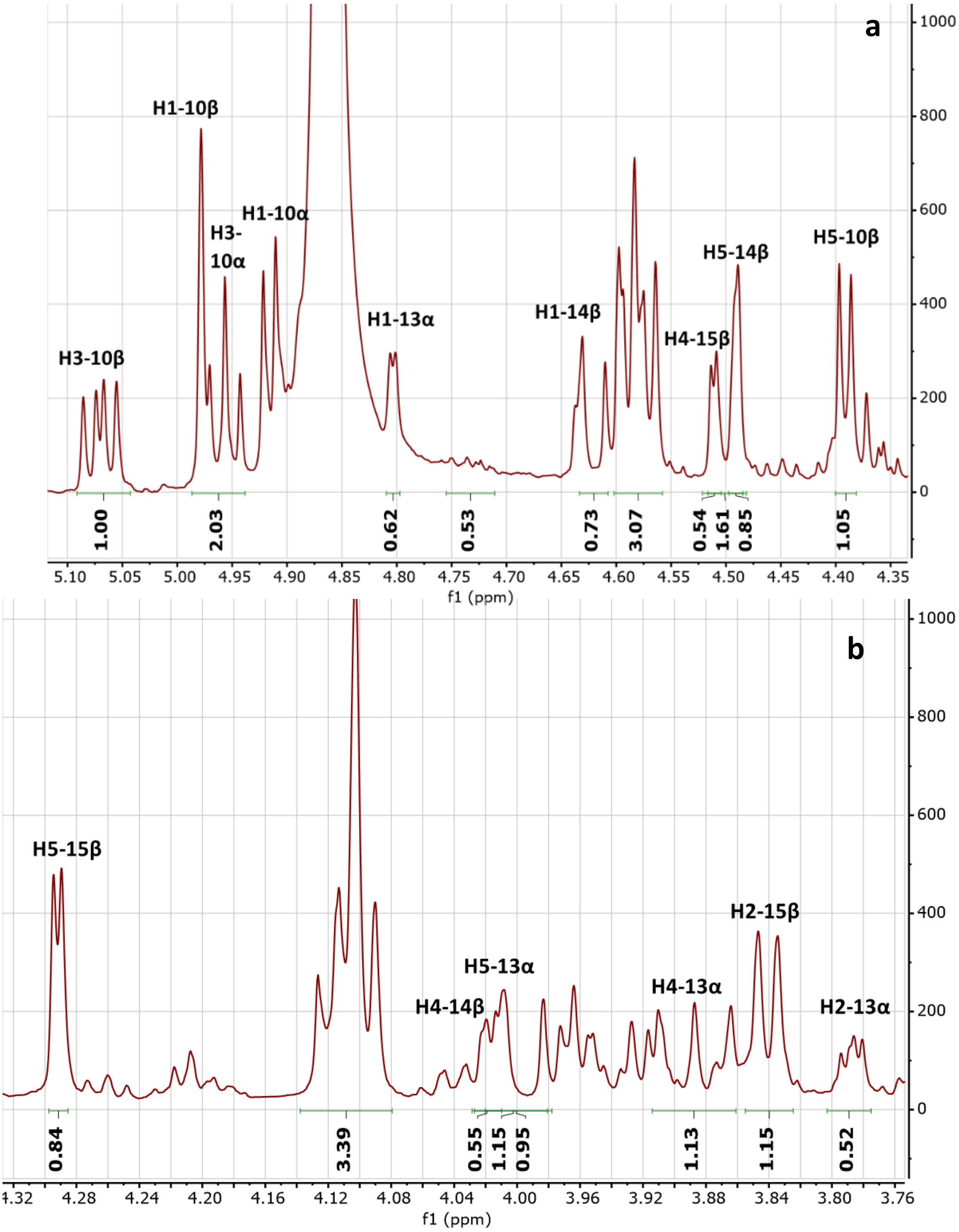

Figure S12: Zooms of ^{1}H NMR spectrum of **H-C$_{12}$ Gul** from OG in the zones: (a) 5.15-4.35 ppm; (b) 4.32-3.76 ppm (400 MHz, CD_3OD).

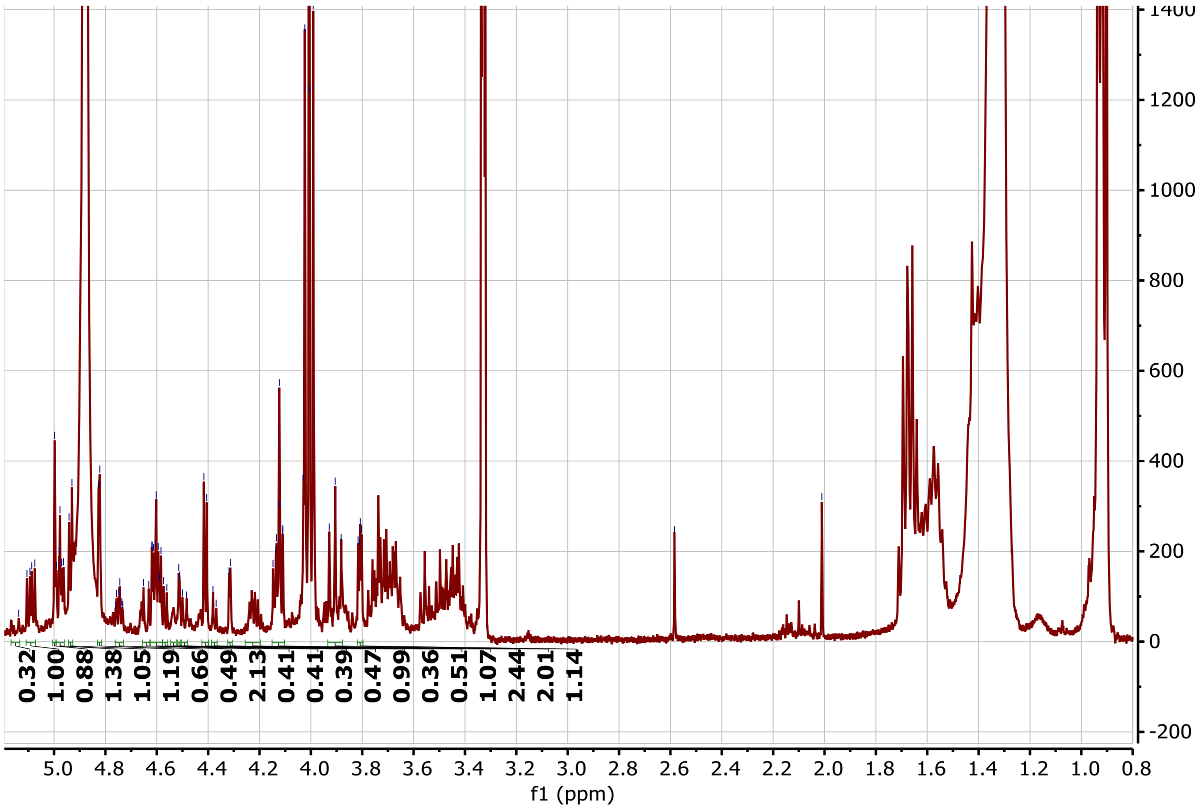

Figure S13: ^{1}H NMR spectrum of **H-C$_{12}$ OAlg** from OAlg (δ: 5.2-0.6 ppm) (400 MHz, CD_3OD).

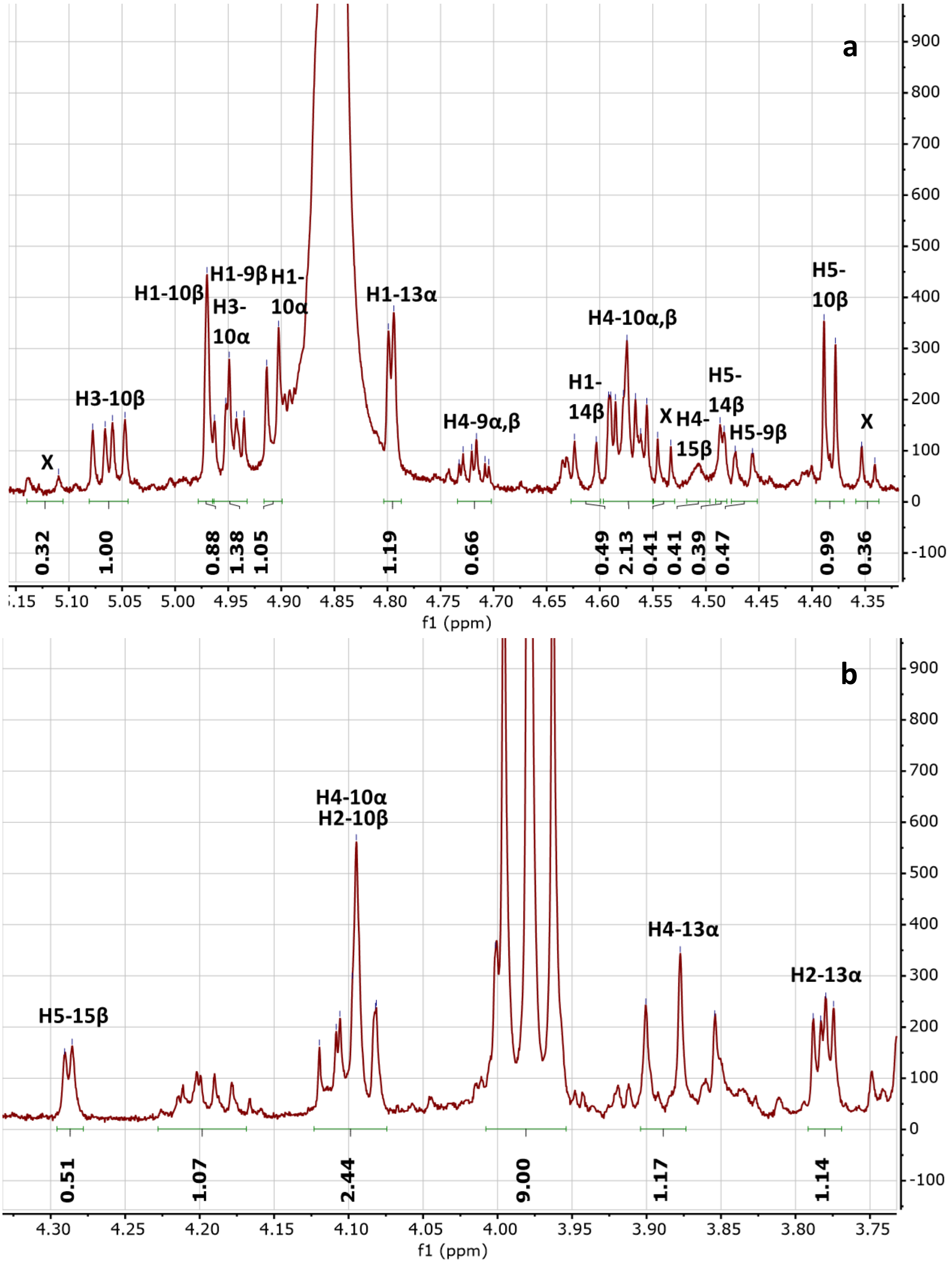

Figure S14: Zooms of ^{1}H NMR spectrum of **H-C_{12} OAlg** from OAlg in the zones: (a) 5.15-4.35 ppm; (b) 4.32-3.74 ppm (400 MHz, CD_3OD).

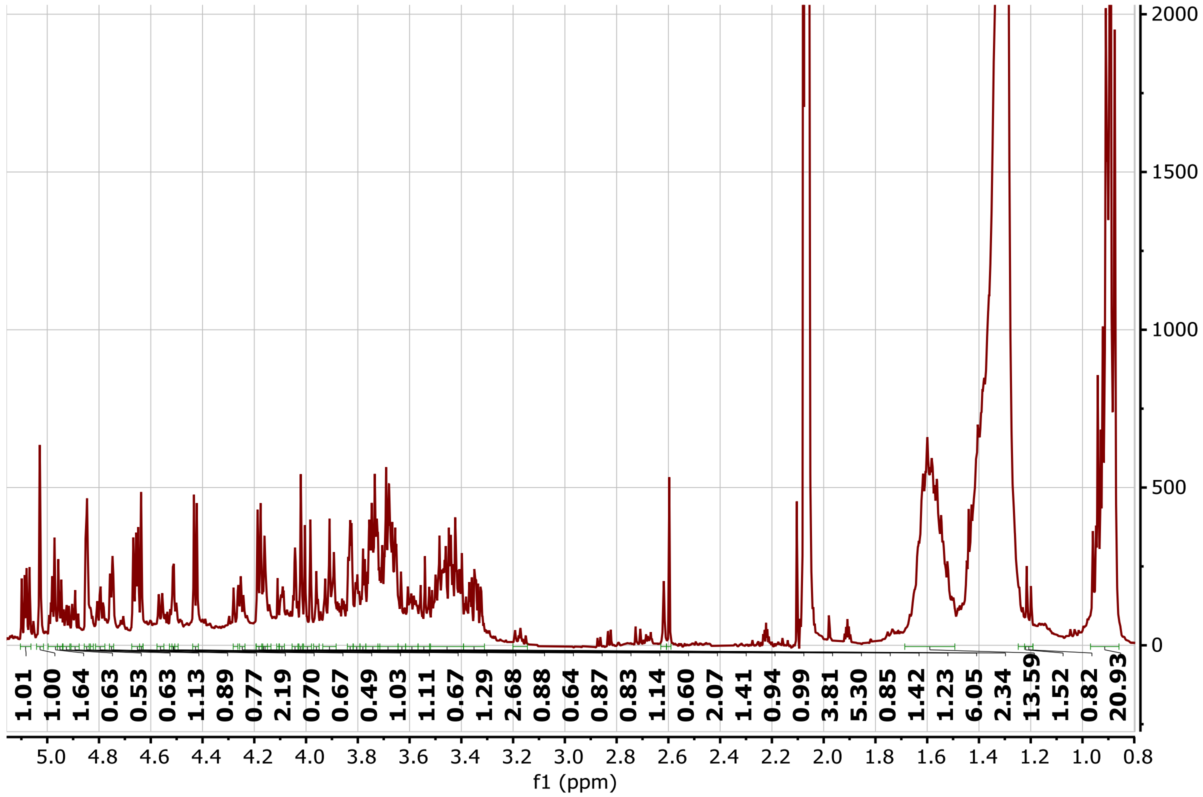

Figure S15: ^{1}H NMR spectrum of **H-C12 s-r Alg** from s-r Alg (δ: 5.2-0.6 ppm) (400 MHz, acetone-d6).

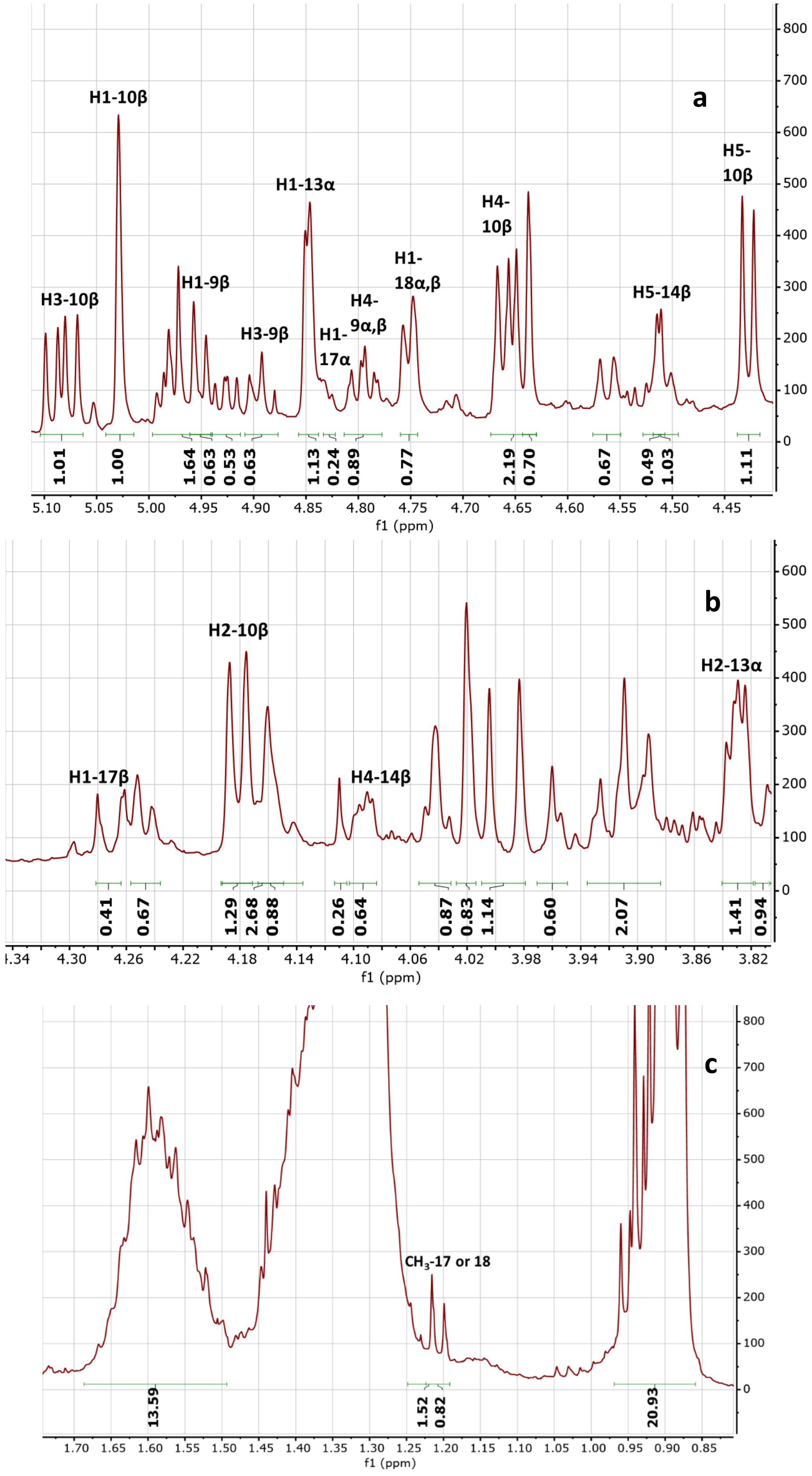

Figure S16: Zooms of ^{1}H NMR spectrum of **H-C12 s-r Alg** from s-r Alg in the zones: (a) 5.10-4.40 ppm; (b) 4.34-3.81 ppm; (c) 1.75-0.80 ppm (400 MHz, acetone-d6).

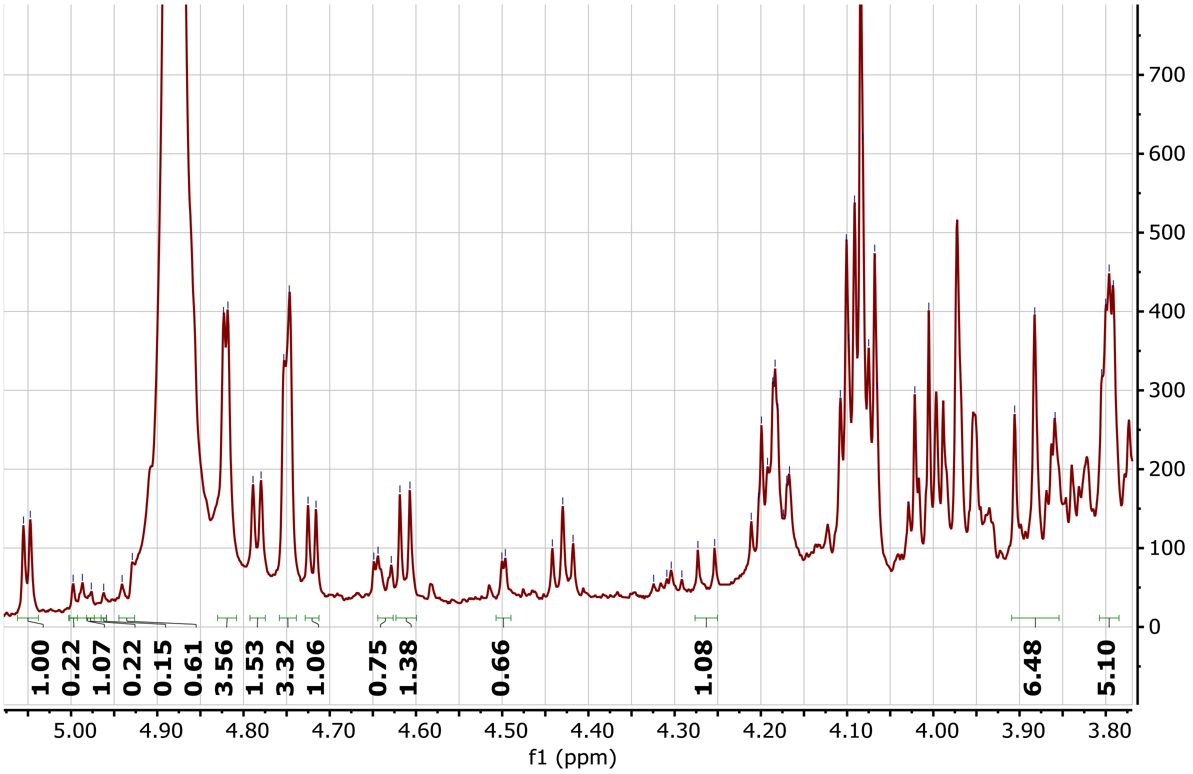

Figure S17: ¹H NMR spectrum of **H-C8 crude Alg** from crude Alg (δ: 5.20-3.80 ppm) (400 MHz, CD_3OD).

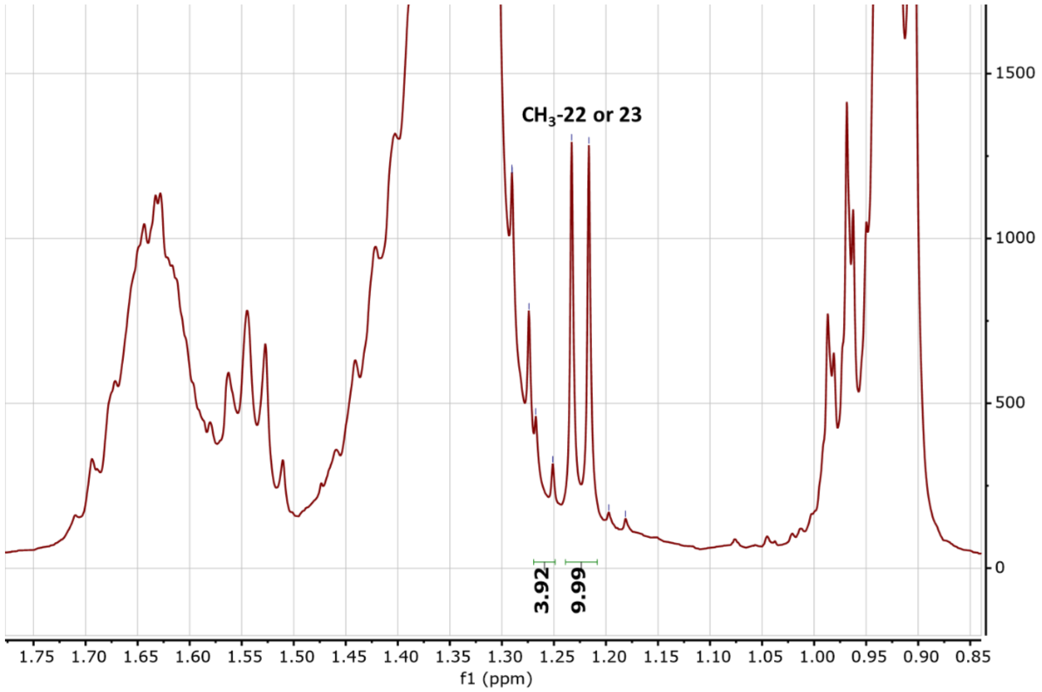

Figure S18: Zoom of ¹H NMR spectrum of **H-C8 crude Alg** from crude Alg in the zone 1.76-0.85 (400 MHz, CD_3OD).

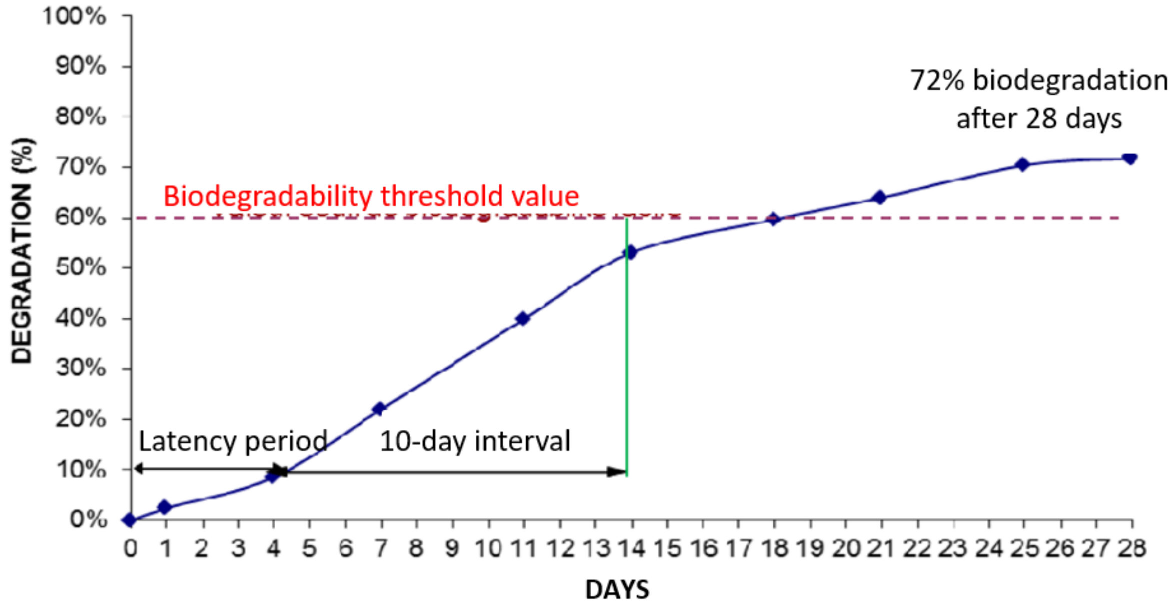

Figure S19: Biodegradability results (**H-C12 s-r Alg**) according to the OCDE 301 B method.

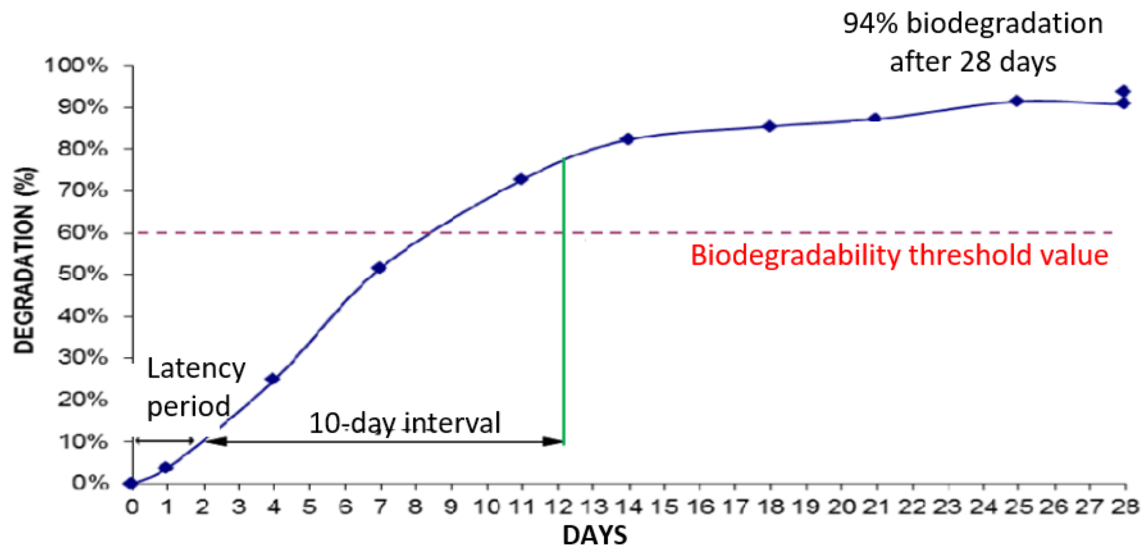

Figure S20: Biodegradability results (**SLES**) according to the OCDE 301 B method.
